# Umbrella Review for Pediatric Sepsis and Robust Prediction Potential Worldwide

**DOI:** 10.1002/pdi3.70069

**Published:** 2026-09-24

**Authors:** Wenbo Fu, Yumei Li, Kee Chong Ng, Sheng Fu

**Affiliations:** ^1^ Lee Kong Chian School of Medicine Nanyang Technological University Singapore; ^2^ Children’s Medicine Center The First Hospital of Jilin University Changchun China; ^3^ KK Women’s and Children’s Hospital Singapore; ^4^ Asia Pacific Advanced Health and Medical Institution Singapore

**Keywords:** antimicrobial resistance, biomarkers, global health, machine learning, pediatric sepsis, prediction models, quality improvement, umbrella review

## Abstract

Pediatric sepsis remains a significant global health burden with considerable mortality and morbidity, particularly in resource‐limited settings. This umbrella review synthesizes current evidence from systematic reviews and meta‐analyses on the definitions, epidemiology, diagnostic approaches, prognostic models, and emerging prediction technologies of pediatric sepsis worldwide. Through comprehensive evaluation of 18 high‐quality systematic reviews published between 2019 and 2024, we identified critical gaps in early recognition and management protocols while highlighting promising technological innovations that demonstrate robust prediction potential. Our analysis revealed substantial variation in sepsis outcomes across different healthcare settings, with approximately 85% of global pediatric sepsis deaths occurring in low‐ and middle‐income countries (LMICs). Quantitative synthesis of prediction models demonstrated pooled sensitivity of 0.81 (95% confidence interval [CI]: 0.76–0.85) and specificity of 0.79 (95% CI: 0.74–0.84), with area under the receiver operating characteristic curve values ranging from 0.78 to 0.94 depending on resource setting and model complexity. The integration of machine learning algorithms with traditional clinical markers showed particular promise for developing context‐appropriate prediction tools that function effectively across resource settings. Etiological analysis revealed distinct pathogen profiles by age and geography: Gram‐negative organisms were predominant in neonates (68%) and LMICs (72%), while Gram‐positive pathogens accounted for 58% of cases in high‐income country settings. Antimicrobial resistance exceeded 50% for common pathogens in LMICs, critically complicating empirical treatment. This review provides a comprehensive foundation for future research directions and implementation strategies to reduce the global burden of pediatric sepsis through precision medicine approaches and health system strengthening.

## 1 Introduction

Sepsis represents a life‐threatening organ dysfunction caused by a dysregulated host response to infection, with children particularly vulnerable to its rapid progression and devastating consequences. Despite advances in critical care medicine, pediatric sepsis continues to impose a substantial global health burden. The latest epidemiological analyses estimate that sepsis contributes to approximately 1.2 million child deaths annually, with disproportionate impact on children in low‐resource settings [1]. The heterogeneity in clinical presentation, diagnostic criteria, and management approaches across diverse healthcare environments complicates the efforts to establish standardized protocols for early recognition and intervention.

This umbrella review aims to synthesize current evidence from systematic reviews and meta‐analyses on the definitions, epidemiology, diagnostic approaches, prognostic models, and emerging prediction technologies of pediatric sepsis worldwide. By critically evaluating high‐quality evidence syntheses published between 2019 and 2024, we seek to identify critical gaps and opportunities for developing robust prediction systems that can be effectively implemented across varied healthcare contexts. Our analysis particularly focuses on the potential for integrating technological innovations with traditional clinical assessment to enhance early detection and improve outcomes for children with sepsis globally, with special attention to resource‐appropriate solutions for high‐burden settings.

## 2 Methods

This umbrella review was conducted according to the Preferred Reporting Items for Systematic reviews and Meta‐Analyses (PRISMA) guidelines for systematic reviews of reviews. The protocol was registered in the International Prospective Register of Systematic Reviews (PROSPERO) with registration number of CRD42024548721.

### 2.1 Eligibility Criteria

We included systematic reviews and meta‐analyses that:

1. Focused on pediatric sepsis (neonates to adolescents ≤ 18 years).
2. Were published in English between January 2019 and October 2024.
3. Addressed at least one of the following key domains: definitions/diagnostic criteria, global epidemiology, biomarkers, prognostic models, prediction technologies, or quality improvement interventions.
4. Used systematic methodology with explicit search strategies and inclusion criteria.
5. Assessed the quality of included primary studies.

### 2.2 Search Strategy and Information Sources

We conducted a comprehensive literature search across four electronic databases (PubMed/MEDLINE, EMBASE, Web of Science, and Cochrane Library) from January 2019 to October 2024. The search strategy combined terms related to pediatric sepsis, systematic reviews, and our specific domains of interest. We also manually screened reference lists of included reviews and contacted experts in the field to identify additional relevant reviews. The complete search strategy is provided in Supporting Information S1: Annex 1.

### 2.3 Study Selection Process

Two investigators screened the titles and abstracts of identified reviews independently against the eligibility criteria. Potentially eligible reviews underwent full‐text assessment by the same investigators independently. Disagreements were resolved through discussion with a third investigator. The study selection process is documented in a PRISMA flow diagram (Supporting Information S1: Annex 2).

### 2.4 Population‐Intervention‐Comparison‐Outcome (PICO) Framework

Our review was structured using the following PICO framework:

- Population: children (neonates to adolescents ≤ 18 years) with suspected or confirmed sepsis or being at risk of developing sepsis in healthcare settings worldwide.
- Intervention/Exposure: diagnostic criteria, prediction models, biomarkers, technological interventions, or quality improvement programs for pediatric sepsis recognition and management.
- Comparison: standard clinical assessment, alternative prediction tools, or historical controls.
- Outcomes: primary outcomes included mortality, time to intervention, and predictive accuracy (sensitivity/specificity); and secondary outcomes included organ dysfunction progression, length of hospital/ICU stay, appropriate antibiotic administration, and resource utilization.

### 2.5 Quality Assessment and Data Extraction

The methodological quality of the included systematic reviews was assessed independently by two investigators using A MeaSurement Tool to Assess systematic Reviews 2 (AMSTAR 2) critical appraisal instrument. Reviews were categorized as of high, moderate, or low quality based on their assessment scores (Supporting Information S1: Annex 3). Data extraction focused on the characteristics of the review, research questions, search strategies, inclusion/exclusion criteria, number and design of included primary studies, quality assessment methods, synthesis approaches, key findings, limitations, and funding sources. For reviews that included meta‐analyses, we extracted effect estimates with confidence intervals [CIs].

### 2.6 Data Synthesis and Analysis

Given the expected heterogeneity in research questions, populations, interventions, and outcomes across the eligible reviews, we conducted a narrative synthesis organized by our key domains of interest. For domains with sufficient comparable data, we performed quantitative synthesis of the most robust findings. We assessed the certainty of evidence across reviews using an adapted GRADE approach for umbrella reviews. Geographical distribution of primary studies within the included reviews was analyzed to identify representation gaps (Supporting Information S1: Annex 4).

## 3 Results

### 3.1 Study Selection and Characteristics

Our initial search yielded 32 potentially eligible systematic reviews and meta‐analyses. After applying inclusion/exclusion criteria and quality assessment, 18 reviews met our final inclusion criteria (see the PRISMA flow diagram in Supporting Information S1: Annex 2). These 18 reviews encompassed data from 367 primary studies involving over 245,000 children with sepsis worldwide. The included reviews were published between 2021 and 2024, with 9 (50%) published in 2023–2024. According to the AMSTAR‐2 assessment, 12 reviews (67%) were rated as high quality, 4 (22%) as moderate quality, and 2 (11%) as low quality (Supporting Information S1: Annex 3).

The geographical distribution of primary studies within the included reviews revealed significant representation imbalances: 68% of primary studies originated from high‐income countries, 18% from upper‐middle income countries, and only 14% from lower‐middle and low‐income countries (Supporting Information S1: Annex 4). This imbalance was particularly pronounced in reviews focusing on prediction technologies and biomarker validation.

### 3.2 Current Definitions and Criteria for Pediatric Sepsis

The 2021 systematic review and meta‐analysis published by the pediatric sepsis definition taskforce established consensus criteria emphasizing life‐threatening organ dysfunction caused by a dysregulated host response to infection [2]. This high‐quality review synthesized evidence from 42 studies involving over 55,000 children to refine the definition of pediatric sepsis to include specific clinical criteria for organ dysfunction in children, moving beyond previous definitions that relied predominantly on systemic inflammatory response syndrome (SIRS) criteria.

The taskforce’s criteria incorporate age‐specific vital sign parameters, laboratory markers of organ dysfunction, and clinical assessment of mental status changes. This standardization effort has enhanced the ability to identify children with sepsis consistently across different healthcare settings, facilitating more accurate epidemiological assessment and enabling more reliable comparison of outcomes across studies [2]. However, implementation challenges persist, particularly in resource‐limited settings where laboratory testing and monitoring capabilities may be constrained.

### 3.3 Global Epidemiology, Etiology, and Burden of Disease

The contemporary epidemiology of pediatric sepsis reveals stark global disparities. Watson et al. [1] documented that while high‐income countries have made significant progress in reducing sepsis mortality, children in low‐ and middle‐income countries (LMICs) continue to experience substantially higher case‐fatality rates. Their analysis revealed that approximately 85% of global pediatric sepsis deaths occur in LMICs, with neonates and children under 5 years bearing the greatest burden.

### 3.4 Etiological Classification by Age Group, Geography, and Resistance Patterns

We conducted a comprehensive synthesis of pathogen profiles across the included reviews, revealing distinct patterns by age group, geographic region, and antimicrobial resistance status (Table 1). This classification addresses critical gaps in understanding context‐specific sepsis etiology necessary for developing appropriate prediction and management strategies.

**TABLE 1:** Etiological classification of pediatric sepsis pathogens.

Age group	High‐income countries	Low‐ and middle‐income countries	Predominant resistance patterns
Neonates (< 28 days)	Gram‐positive: 42% (GBS 28%, *Staphylococcus aureus [S. aureus]* 14%) Gram‐negative: 38% (*Escherichia coli [E. coli]* 25%, *Klebsiella* 13%) Fungal: 8%	Gram‐negative: 68% (*Klebsiella* 32%, *E. coli* 24%, *Pseudomonas* 12%) Gram‐positive: 24% (*S. aureus* 18%, GBS 6%) Fungal: 5%	ESBL production: 58% of Gram‐negative isolates Carbapenem resistance: 22% in *Klebsiella* MRSA: 35% of *S. aureus* isolates
Infants (1–12 months)	Viral: 45% (RSV, influenza) Gram‐positive: 30% (*Streptococcus pneumoniae [S. pneumoniae]* 18%, *S. aureus* 12%) Gram‐negative: 20%	Gram‐negative: 52% (*Salmonella* 22%, *E. coli* 18%, *Klebsiella* 12%) Gram‐positive: 33% (*S. pneumoniae* 20%, *S. aureus* 13%) Viral: 12%	MDR *Salmonella*: 48% in sub‐Saharan Africa Penicillin‐resistant *S. pneumoniae*: 32% ESBL *E. coli*: 45%
Children (1–12 years)	Gram‐positive: 58% (*S. pneumoniae* 35%, *S. aureus* 23%) Viral: 28% Gram‐negative: 12%	Gram‐negative: 48% (*Salmonella* 25%, *E. coli* 15%, *Shigella* 8%) Gram‐positive: 38% (*S. pneumoniae* 24%, *S. aureus* 14%) Parasitic: 8% (malaria co‐infection)	MDR *Salmonella* *Typhi*: 65% in South Asia Ceftriaxone‐resistant *S. pneumoniae*: 28% Artemisinin‐resistant malaria co‐infection: 18% in Southeast Asia
Adolescents (13–18 years)	Gram‐positive: 62% (*S. aureus* 42%, *S. pneumoniae* 20%) Gram‐negative: 25% Fungal: 8%	Gram‐negative: 45% (*Salmonella* 20%, *E. coli* 15%, *Klebsiella* 10%) Gram‐positive: 40% (*S. aureus* 32%, *S. pneumoniae* 8%) Mycobacterial: 10%	MRSA: 52% of *S. aureus* isolates ESBL production: 55% of Gram‐negative isolates MDR‐TB co‐infection: 15% in high‐burden settings

Abbreviations: ESBL, extended‐spectrum beta‐lactamase; MDR‐TB, multidrug‐resistant tuberculosis; MRSA, Methicillin‐resistant *Staphylococcus aureus*.

These geographical variations underscore the need for context‐specific prediction and management strategies that account for local epidemiology, available resources, and healthcare infrastructure. Critically, antimicrobial resistance patterns significantly complicate empirical treatment approaches in LMICs, where resistance to first‐line antibiotics exceeds 50% for common pathogens [1, 3]. This resistance burden necessitates prediction tools that incorporate local resistance patterns into risk stratification algorithms.

### 3.5 Diagnostic Approaches and Biomarkers

#### 3.5.1 Quantitative Synthesis of Biomarker Performance

We performed meta‐analytic pooling of biomarker performance data where sufficient homogeneity existed across primary studies within the included reviews. C‐reactive protein (CRP) demonstrated pooled sensitivity of 0.76 (95% CI: 0.71–0.80) and specificity of 0.78 (95% CI: 0.73–0.82) for neonatal sepsis detection in LMICs [4]. Procalcitonin (PCT) showed comparable performance with pooled sensitivity of 0.72 (95% CI: 0.67–0.77) and specificity of 0.81 (95% CI: 0.76–0.85). Interleukin‐6 (IL‐6) demonstrated higher sensitivity (0.84, 95% CI: 0.78–0.88) but lower specificity (0.69, 95% CI: 0.63–0.74), making it potentially valuable for ruling out sepsis when negative. Combination biomarker strategies (CRP + PCT) significantly improved diagnostic accuracy with pooled area under the receiver operating characteristic curve (AUC) of 0.89 (95% CI: 0.85–0.92) compared to single biomarkers (AUC 0.78–0.83) [4, 5].

The diagnosis of pediatric sepsis remains challenging due to non‐specific clinical presentations that often overlap with non‐infectious inflammatory conditions. Biomarkers have emerged as valuable adjuncts to clinical assessment, with CRP, PCT, IL‐6, and presepsin showing varying degrees of diagnostic accuracy [4, 5].

Rees et al. [4] conducted a systematic review and meta‐analysis of 42 studies involving 8754 neonates specifically examining biomarker performance in LMICs. Their analysis revealed that while CRP and PCT demonstrated moderate diagnostic accuracy (pooled sensitivity 0.76 and 0.72, respectively; specificity 0.78 and 0.81, respectively), their performance was significantly influenced by timing of measurement, gestational age, and local pathogen profiles. Wong [5] further emphasized that biomarker utility extends beyond diagnosis to prognostic enrichment, with combinations of biomarkers showing greater promise than single markers for risk stratification.

However, biomarker implementation faces significant challenges in resource‐limited settings where laboratory infrastructure may be inadequate and costs prohibitively. This reality necessitates the development of prediction tools that minimize dependence on laboratory testing while maintaining high sensitivity for early sepsis detection.

### 3.6 Prognostic Models in Diverse Healthcare Settings

#### 3.6.1 Quantitative Synthesis of Prediction Model Performance

We synthesized performance metrics across prediction models stratified by resource setting (Table 2). In LMIC settings, clinical prediction models incorporating 4–6 readily available parameters achieved pooled sensitivity of 0.81 (95% CI: 0.76–0.85) and specificity of 0.79 (95% CI: 0.74–0.84), with AUC values ranging from 0.78 to 0.88. Technology‐enhanced models in high‐resource settings demonstrated superior discrimination with pooled AUC of 0.91 (95% CI: 0.88–0.94), but their implementation feasibility in LMICs remains limited by infrastructure requirements.

**TABLE 2:** Quantitative synthesis of prediction model performance by resource setting.

Resource setting	Model type	Number of models	Pooled sensitivity (95% CI)	Pooled specificity (95% CI)	Pooled AUC (95% CI)	Key limiting factors
LMIC (primary/secondary care)	Clinical signs only	7	0.83 (0.78–0.87)	0.76 (0.71–0.81)	0.84 (0.80–0.88)	Limited validation outside development setting; variable inter‐rater reliability
LMIC (tertiary care)	Clinical + basic labs	5	0.79 (0.73–0.84)	0.82 (0.77–0.86)	0.85 (0.81–0.89)	Laboratory infrastructure constraints; supply chain interruptions for reagents
High‐income countries	EHR‐based alerts	6	0.86 (0.82–0.90)	0.81 (0.77–0.85)	0.90 (0.87–0.93)	Alert fatigue (false positive rate 32%–45%); workflow disruption
High‐income countries	AI/ML‐enhanced	4	0.91 (0.87–0.94)	0.87 (0.83–0.91)	0.93 (0.90–0.96)	Limited external validation; algorithmic bias; data privacy concerns

Abbreviations: AI, artificial intelligence; AUC, area under the receiver operating characteristic curve; CI, confidence interval; EHR, electronic health record; LMIC, low‐ and middle‐income country/countries; ML, machine learning.

### 3.7 Real‐World Implementation Failures in LMIC Settings: Critical Analysis

Despite promising performance metrics in controlled studies, multiple prediction tools have failed during real‐world implementation in LMICs. Our synthesis identified four critical failure modes:

1. Infrastructure–technology mismatch: In a multi‐center implementation across 12 district hospitals in Kenya and Uganda, an electronic health record (EHR)‐based early warning system achieved only 28% adherence due to unreliable electricity (power outages averaging 4.2 hours/day), intermittent internet connectivity (available < 6 hours/day in 67% of facilities), and insufficient computer terminals (1 terminal per 45 beds vs. required 1:8 ratio) [6]. Mortality outcomes showed no improvement compared to standard care (odds ratio [OR] 0.94, 95% CI: 0.78–1.13).
2. Workflow disruption without process redesign: Implementation of a mobile health sepsis recognition app in 8 rural clinics in Bangladesh initially showed 76% uptake among healthcare workers. However, within 6 months, usage declined to 23% because the app required 8–12 minutes per assessment, which was not accounted for in existing workflows. Clinicians reported that the tool created additional documentation burden without streamlining care processes or providing clear escalation pathways [6]. No mortality reduction was observed (absolute risk reduction 0.8%, 95% CI: −2.1% to 3.7%).
3. Task‐shifting without adequate training: A simplified sepsis recognition protocol was implemented across 15 primary health centers in Malawi with task‐shifting to community health workers (CHWs). Despite high initial training completion rates (92%), 6‐month competency assessments revealed that only 38% of CHWs could correctly identify all critical signs of sepsis. This was associated with inappropriate antibiotic use in 42% of suspected cases and delayed referrals in 29% of true sepsis cases. The intervention paradoxically increased mortality in children under 1 year (adjusted OR 1.34, 95% CI: 1.08–1.67) due to delayed appropriate care [6].
4. Context‐inappropriate threshold calibration: A prediction model developed in a South African tertiary hospital was implemented without recalibration across 7 district hospitals in rural settings. The model’s threshold for “high risk” was based on the availability of resources (intensive care unit beds, vasopressors, and mechanical ventilation) at the site the model was developed in. In district settings lacking these resources, 68% of children flagged as “high risk” could not receive the indicated interventions, leading to frustration among clinicians, alert dismissal, and ultimately abandonment of the tool after 4 months [6, 7].

These failures highlight that prediction tool accuracy alone is insufficient for successful implementation; contextual adaptation addressing infrastructure realities, workflow integration, intensity of appropriate training, and resource‐aligned risk stratification is essential for real‐world impact.

Recent evidence has focused on developing and validating prognostic models specifically for children with sepsis in resource‐limited environments. Jordan et al. [7] conducted a systematic review and meta‐analysis of 15 prognostic models in children with sepsis in LMICs, identifying significant gaps in model development and validation. Their analysis revealed that most existing models were developed in high‐income settings and demonstrated poor generalizability to LMIC contexts where resource constraints, pathogen profiles, and comorbidities differ substantially.

The review highlighted several promising approaches that incorporate readily available clinical parameters rather than laboratory values, making them more feasible for implementation in resource‐limited settings. Notable examples include modifications of the WHO emergency triage assessment and treatment guidelines and simplified versions of the pediatric index of mortality adapted for low‐resource environments [3]. However, the authors emphasized that rigorous external validation across diverse settings remains a critical gap in the literature. This finding is supported by a multi‐country external validation study in sub‐Saharan Africa, which found that commonly used pediatric prognostic models exhibited poor calibration and discrimination when applied to local populations, with AUC values dropping by 0.12–0.24 compared to the cohorts where they were originally developed [8].

Detailed performance metrics of these prediction models are provided in Supporting Information S1: Annex 5.

### 3.8 Prediction Technologies and Digital Health Solutions

#### 3.8.1 Critical Analysis of AI‐Related Risks in Sepsis Prediction

Although AI‐driven prediction technologies show promise, our synthesis identified four critical risk domains requiring careful mitigation before widespread deployment:

1. Algorithmic bias and health inequity: 92% of AI based sepsis prediction models were developed and validated exclusively using data from high‐income countries [9]. When these models were tested on datasets from LMICs, performance declined substantially (AUC reduction of 0.12–0.24 points) due to differences in pathogen profiles, comorbidities (e.g., malnutrition and HIV), and clinical presentation patterns [7, 9]. This risks perpetuating health inequities by deploying tools that work well for privileged populations while failing in high‐burden settings. Federated learning approaches show promise for mitigating this bias but remain underutilized (only 2 of 31 reviewed technologies employed this approach) [9].
2. Alert fatigue and clinical desensitization: EHR‐based sepsis alerts demonstrate high false positive rates (32%–45% across studies), leading to alert fatigue. In a prospective study across 5 children’s hospitals in the United States, clinicians overrode 68% of sepsis alerts by month 6 of implementation, with override rates reaching 89% for low‐acuity patients [9]. Critically, this desensitization extended to true positive alerts, with response time to genuine sepsis cases increasing by 18 minutes (95% CI: 12–24 minutes) after 6 months of alert exposure compared to pre‐implementation baseline.
3. Data privacy and security vulnerabilities: AI systems requiring continuous data streaming create significant privacy risks, particularly for children. Our review identified that only 3 of 31 prediction technologies had undergone formal security auditing, and none addressed re‐identification risks from aggregated clinical data streams [9]. In LMIC settings with weaker data protection frameworks, these vulnerabilities are amplified, potentially exposing vulnerable pediatric populations to privacy breaches.
4. Over‐reliance and clinical deskilling: Emerging evidence suggests that over‐dependence on algorithmic decision support may erode clinical diagnostic skills. A simulation study with pediatric residents demonstrated that those trained primarily with AI decision support showed 23% slower recognition times for atypical sepsis presentations when the AI tool was unavailable compared to traditionally trained peers [9]. This “automation bias” poses particular risks in resource‐limited settings where technology failures are common and clinical judgment remains essential.

These risks necessitate a precautionary approach to AI deployment: rigorous external validation across diverse settings, human‐centered design prioritizing clinician autonomy, transparent reporting of limitations, and continuous monitoring for unintended consequences.

The emergence of digital health technologies offers unprecedented opportunities for enhancing sepsis prediction and early warning systems. Tennant et al. [9] conducted a comprehensive scoping review of 31 digital health technologies for pediatric sepsis prediction, identifying several promising approaches:

1. EHR‐based alert systems: These systems continuously analyze vital signs, laboratory results, and clinical documentation to identify patterns suggestive of early sepsis. Their performance varies significantly based on algorithm sophistication and integration with clinical workflows.
2. Machine learning and artificial intelligence applications: Advanced algorithms that incorporate multiple data streams demonstrate superior performance compared to traditional rule‐based systems. Deep learning approaches that analyze complex temporal patterns in vital signs show particular promise, with AUC values ranging from 0.85 to 0.94 in validation studies.
3. Point‐of‐care technologies: Portable devices capable of rapid biomarker measurement at the bedside may bridge the gap between high‐resource prediction tools and LMIC feasibility requirements.
4. Mobile health applications: Smartphone‐based tools that guide clinical assessment and risk stratification show potential for scaling the recognition of sepsis in community and primary care settings.

However, the review noted significant challenges in implementation, including algorithm bias, integration with existing workflows, and lack of validation in diverse populations [9]. The authors emphasized that technological solutions must be developed with consideration for local context, infrastructure limitations, and healthcare worker capabilities. Importantly, 92% of the prediction technologies reviewed had been developed and validated exclusively in high‐income countries, limiting their generalizability to resource‐constrained settings.

### 3.9 Quality Improvement Programs and Implementation Science

Effective prediction tools must be integrated within comprehensive quality improvement frameworks to achieve meaningful impact on outcomes. De Souza et al. [6] examined 28 quality improvement programs in pediatric sepsis from a global perspective, identifying core components that drive success across diverse settings:

1. Standardized protocols with local adaptation: Evidence‐based guidelines modified to address resource constraints and local pathogen profiles.
2. Multidisciplinary team engagement: Involvement of physicians, nurses, pharmacists, and laboratory staff in protocol development and implementation.
3. Education and simulation training: Context‐appropriate training materials and regular simulation exercises to reinforce recognition and response skills.
4. Data‐driven feedback mechanisms: Simple audit tools that provide regular feedback on process and outcome metrics.
5. Family engagement strategies: Approaches to incorporate caregiver observations and concerns into early warning systems.

The analysis revealed that successful programs in LMICs often focused on simplifying protocols, leveraging existing infrastructure, and training non‐physician healthcare workers to recognize and initiate sepsis management [6]. The most effective programs demonstrated 25%–40% reductions in sepsis mortality when prediction tools were integrated with comprehensive quality improvement frameworks. These insights provide valuable guidance for implementing prediction technologies within practical quality improvement frameworks.

### 3.10 Robust Prediction Potential: Integrating Approaches for Global Impact

The synthesis of current evidence suggests that robust prediction systems for pediatric sepsis must integrate multiple approaches tailored to specific healthcare contexts:

1. Tiered prediction models: Systems that function effectively across different resource levels, with basic models for primary care settings and more sophisticated versions for tertiary centers. For example, a simplified clinical model using respiratory rate, temperature, and mental status could be deployed in community settings, while tertiary hospitals might implement integrated models incorporating vital sign trends, laboratory markers, and AI‐based pattern recognition.
2. Hybrid algorithms: Approaches that combine clinical assessment parameters with selective biomarker testing and technological monitoring where available. Jordan et al. [7] demonstrated that hybrid models incorporating clinical signs with one or two targeted biomarkers achieved comparable accuracy to complex laboratory‐dependent models while being more feasible in resource‐limited settings.
3. Context‐adaptive machine learning: Algorithms trained on diverse global datasets that account for regional variations in presentation, pathogens, and available resources. Tennant et al. [9] highlighted emerging federated learning approaches that enable model training across multiple institutions without sharing raw patient data, potentially addressing privacy concerns while improving generalizability.
4. Implementation‐focused design: Prediction tools developed with direct input from end‐users in target settings to ensure practical utility and workflow integration. De Souza et al. [6] emphasized that successful implementation requires addressing not just technical accuracy but also human factors, including alert fatigue mitigation and clear escalation protocols.

Hibberd et al. [10] emphasized that recognizing sepsis in children’s emergency care requires balancing sensitivity and specificity while accounting for the rapid progression potential in pediatric patients. Their clinical practice review highlights the importance of serial assessment and vital sign trends over single‐point evaluations, suggesting that robust prediction systems must incorporate temporal dynamics rather than static assessments. They recommend that high‐sensitivity prediction tools should be deployed at initial triage, followed by more specific assessments as clinical information accumulates.

### 3.11 Challenges and Future Directions

Despite significant advances in understanding and managing pediatric sepsis, several critical challenges remain:

1. Resource disparity: The gap between sophisticated prediction technologies developed in high‐income settings and the practical realities of LMIC healthcare facilities. Over 90% of AI‐based sepsis prediction research originates from high‐income countries, with minimal validation in resource‐constrained settings [9].
2. Validation gaps: Limited external validation of prediction models across diverse populations and settings. Jordan et al. [7] found that only 2 of 15 prognostic models for children with sepsis in LMICs had undergone external validation outside the setting where they were developed.
3. Implementation barriers: Challenges in integrating new technologies with existing clinical workflows and overcoming resistance to algorithmic decision support. De Souza et al. [6] identified workflow integration as the most common failure point in otherwise promising quality improvement programs.
4. Data infrastructure limitations: Inadequate digital infrastructure in many regions limiting the deployment of advanced prediction systems. Only 18% of healthcare facilities in low‐income countries have the necessary digital infrastructure to support EHR‐based prediction tools [9].
5. AI‐specific risks: Algorithmic bias perpetuating health inequities, alert fatigue leading to desensitization, data privacy vulnerabilities, and potential clinical deskilling due to over‐reliance on technology—all requires proactive mitigation strategies before widespread deployment.

### 3.12 Recommendations for Future Research and Implementation

Future research priorities should focus on:

- Developing and validating context‐specific prediction models for LMIC settings through global collaborative networks with equitable data sharing.
- Creating simplified low‐cost technologies that maintain high predictive value while addressing resource constraints.
- Establishing mandatory external validation requirements across diverse geographic and resource settings before clinical deployment of AI tools.
- Implementing bias detection and mitigation frameworks during AI model development, including diverse training datasets and fairness metrics.
- Incorporating local pathogen profiles and antimicrobial resistance patterns into prediction algorithms to enhance clinical utility.
- Investigating implementation strategies that account for healthcare system constraints, particularly focusing on sustainable task‐shifting models with competency maintenance.
- Conducting longitudinal studies on unintended consequences of AI deployment, including effects on clinical skills, equity, and workflow dynamics.
- Developing regulatory frameworks specific to pediatric AI applications that address privacy, safety, and equity concerns.

## 4 Conclusion

Pediatric sepsis remains a formidable global health challenge requiring sophisticated yet practical approaches to early recognition and intervention. This umbrella review synthesized current evidence from 18 high‐quality systematic reviews and meta‐analyses, demonstrating significant progress in standardizing definitions, understanding epidemiology, and developing prediction technologies related to pediatric sepsis. However, substantial gaps persist between technological potential and practical implementation, particularly in resource‐limited settings where the burden of disease is greatest.

Our quantitative synthesis demonstrates that context‐appropriate prediction models can achieve robust performance (pooled sensitivity 0.81 and specificity 0.79) even in resource‐constrained settings when designed with implementation realities in mind. The etiological classification revealed critical pathogen and resistance pattern variations that must inform context‐specific prediction tool development. However, real‐world implementation failures demonstrated that technical accuracy alone is insufficient: successful deployment requires addressing infrastructure limitations, workflow integration, appropriate training, and resource‐aligned risk stratification.

Critically, the rapid adoption of AI‐driven prediction tools demands cautious optimism. Although these technologies show promise, significant risks—including algorithmic bias (AUC reduction 0.12–0.24 in LMIC settings), alert fatigue (68% override rates by month 6), privacy vulnerabilities (only 3 of 31 tools audited for security), and potential clinical deskilling (23% slower recognition of atypical presentations)—require proactive mitigation before widespread deployment. The path forward requires balancing technological innovation with implementation pragmatism, ensuring that advances benefit all children regardless of geography or socioeconomic status.

By integrating precision medicine approaches with pragmatic quality improvement strategies and prioritizing equity in technology development, the global health community can make meaningful progress toward reducing pediatric sepsis mortality and morbidity worldwide. Future efforts must prioritize developing adaptable prediction tools that function effectively across the spectrum of healthcare settings while building sustainable capacity for their implementation. The ultimate goal must be equitable access to life‐saving recognition and response systems for sepsis for all children globally.

## Author Contributions

**Wenbo Fu:** conceptualization, data curation, formal analysis, investigation, methodology, writing – original draft, writing – review and editing. **Yumei Li:** data curation, formal analysis, investigation, validation, visualization, writing – review and editing. **Kee Chong Ng:** conceptualization, methodology, resources, supervision, writing – review and editing. **Sheng Fu:** conceptualization, methodology, project administration, resources, supervision, writing – original draft, writing – review and editing.

## Funding

The authors have nothing to report.

## Conflicts of Interest

The authors declare no conflicts of interest.

## Supporting information


Supporting Information S1

## Data Availability

The data that supports the findings of this study are available in the supplementary material of this article.
