# Correction to “Umbrella Review for Pediatric Sepsis and Robust Prediction Potential Worldwide”

**DOI:** 10.1002/pdi3.70080

**Published:** 2026-11-03

W. Fu, Y. Li, S. Fu, “Umbrella Review for Pediatric Sepsis and Robust Prediction Potential Worldwide,” *Pediatric Discovery* 4 (2026): e70069, https://doi.org/10.1002/pdi3.70069.

In the originally published article, the author list and affiliation information were incorrect. Kee Chong Ng was listed as an author and has been removed from the author list. The affiliation “KK Women's and Children's Hospital, Singapore” was included and has been removed. The corrected author list is:

Wenbo Fu^1^, Yumei Li^2^ and Sheng Fu^3^

The corrected affiliations are as follows:

^1^Lee Kong Chian School of Medicine, Nanyang Technological University, Singapore

^2^Children's Medicine Center, The First Hospital of Jilin University, Changchun, China

^3^Asia Pacific Advanced Health and Medical Institution, Singapore

The Author Contributions section has been updated accordingly. Professor Kee Chong Ng should instead be acknowledged in the Acknowledgments section, which has been updated to read as given below:

We gratefully acknowledge Professor Kee Chong Ng for contributing the initial idea and suggestions, revising the draft, and providing comments and suggestions on a specific PICO, among other contributions.

We apologize for this error.

