# Supplementary material for: Umbrella Review for Pediatric Sepsis and Robust Prediction Potential Worldwide
Source: Pediatr Discov. 2026 Sep 24;4(3):e70069. doi: 10.1002/pdi3.70069 (PMC13612974; doi:10.1002/pdi3.70069)
Supplement: Supplementary file 1 [file PDI3-4-e70069-s001.docx]

**Supplementary Annexes**

**Annex 1: Search Strategy**

"pediatric"[MeSH Terms] OR "paediatric"[Title/Abstract] OR "child*"[Title/Abstract] OR "neonat*"[Title/Abstract] OR "infant*"[Title/Abstract] OR "adolescen*"[Title/Abstract]) AND ("sepsis"[MeSH Terms] OR "septic shock"[Title/Abstract] OR "systemic inflammatory response syndrome"[Title/Abstract]) AND ("systematic review"[Publication Type] OR "meta-analysis"[Publication Type] OR "systematic review"[Title/Abstract] OR "meta-analysis"[Title/Abstract]) AND ("2019/01/01"[Date - Publication] : "2024/10/31"[Date - Publication]

**Annex 2: PRISMA Flow Diagram**

Figure1. PRISMA Flow Diagram of Study Selection Process

**Annex 3: AMSTAR-2 Quality Assessment of Included Reviews**

**Table A1-1: AMSTAR-2 Critical Appraisal of Included Systematic Reviews**

| Review Citation | Overall Confidence Rating | Critical Domain Assessment (Items 2, 4, 9, 11) | Key Strengths | Key Limitations |
| --- | --- | --- | --- | --- |
| **Menon et al. 2021** Crit Care Med. 2021; 50(1): 21-36 | High | • Item 2: Protocol registered (PROSPERO CRD42019135765) • Item 4: Comprehensive search across 8 databases • Item 9: Used validated tool (modified QUADAS-2) • Item 11: Detailed exclusion justification provided | • International expert panel involvement • Rigorous consensus methodology (modified Delphi) • Comprehensive subgroup analyses by age groups • Detailed assessment of publication bias using funnel plots • Transparent reporting of funding sources and conflicts | • Limited assessment of non-English literature • Some heterogeneity in primary study definitions |
| **Tennant et al. 2024** npj Digit Med. 2024; 7: 353 | High | • Item 2: Protocol registered (PROSPERO CRD42022304215) • Item 4: Systematic search across 9 databases including grey literature • Item 9: Used mixed-methods appraisal tool for diverse study designs • Item 11: Clear exclusion criteria with reasons documented | • Inclusion of both published and unpublished literature • Detailed technical assessment of prediction algorithms • Contextual analysis of implementation barriers • Expert validation of findings • Transparent reporting of search strategy | • Limited assessment of commercial prediction tools not in literature • Heterogeneous outcome measures across included studies |
| **Jordan et al. 2024** Front Pediatr. 2024; 12: 1463986 | High | • Item 2: Protocol registered (PROSPERO CRD42023416790) • Item 4: Comprehensive search strategy with hand-searching • Item 9: Quality assessment using PROBAST tool • Item 11: Detailed exclusion documentation with flow diagram | • Focus on underrepresented LMIC settings • Assessment of model calibration and clinical utility • Detailed contextual analysis of resource constraints • Risk of bias assessment for prediction model studies • External validation assessment | • Limited number of high-quality prognostic studies from LMICs • Statistical heterogeneity in meta-analysis components |
| **de Souza et al. 2024** Lancet Child Adolesc Health. 2024; 8(9): 695-706 | Moderate | • Item 2: Protocol registered • Item 4: Comprehensive search but limited grey literature • Item 9: Quality assessment performed but tool not validated • Item 11: Exclusion criteria documented | • Global representation of authors and contexts • Integration of both quantitative and qualitative evidence • Detailed implementation framework development • Expert consensus validation of findings • Practical recommendations for different resource settings | • Limited assessment of publication bias • Heterogeneous quality of included primary studies • Some subjectivity in synthesis methodology |
| **Hibberd et al. 2025** Pediatr Med. 2025; 8: 11 | Moderate | • Item 2: No registered protocol • Item 4: Limited search strategy (4 databases) • Item 9: Limited risk of bias assessment • Item 11: Basic exclusion documentation | • Clinical practice focus with actionable recommendations • Expert clinical validation by international panel • Clear presentation of key decision points • Integration of recent guideline updates • Practical implementation considerations | • Narrative review components limit systematic rigor • Limited assessment of study quality • Potential author selection bias |
| **Watson et al. 2024** Lancet Child Adolesc Health. 2024; 8(9): 670-681 | High | • Item 2: Protocol registered (PROSPERO CRD42022115783) • Item 4: Comprehensive global search strategy • Item 9: Risk of bias assessment using modified tools • Item 11: Detailed exclusion justification | • Global representation of data sources • Detailed regional and income-level stratification • Assessment of temporal trends in epidemiology • Rigorous statistical analysis of mortality data • Transparent handling of missing data | • Limited primary data from conflict-affected regions • Challenges in standardizing cause-of-death attribution across settings |
| **Rees et al. 2023** BMJ Paediatr Open. 2023; 7(1): e001627 | High | • Item 2: Protocol registered (PROSPERO CRD42021254321) • Item 4: Comprehensive search across 7 databases • Item 9: QUADAS-2 assessment for diagnostic studies • Item 11: Detailed exclusion documentation | • Focus on resource-limited settings • Detailed assessment of biomarker timing effects • Subgroup analyses by gestational age and pathogen profile • Assessment of publication bias • Transparent reporting of limitations | • Limited studies with standardized biomarker protocols • Heterogeneity in reference standards across included studies |
| **Wong 2021** Pediatr Res. 2021; 91(2): 283-288 | Moderate | • Item 2: No registered protocol • Item 4: Limited search strategy (5 databases) • Item 9: Basic quality assessment performed • Item 11: Limited exclusion documentation | • Expert authorship with extensive clinical experience • Clear clinical context for biomarker interpretation • Integration of biological plausibility with clinical data • Practical recommendations for clinical implementation • Clear presentation of biomarker trajectories | • Narrative review components limit systematic rigor • Limited assessment of publication bias • Potential dated evidence (focused on pre-2020 data) |
| **Al-Wassia et al. 2023** JAMA Pediatr. 2023; 177(5): 489-498 | High | • Item 2: Protocol registered (PROSPERO CRD42022275412) • Item 4: Comprehensive search across 9 databases • Item 9: ROBINS-I and ROB2 tools used • Item 11: Detailed exclusion justification | • Individual patient data meta-analysis components • Detailed assessment of time-to-antibiotic effects • Comprehensive sensitivity analyses • Assessment of publication bias using multiple methods • Transparent reporting of funding sources | • Limited representation from sub-Saharan Africa • Heterogeneity in antibiotic protocols across included studies |
| **Balamuth et al. 2022** Pediatrics. 2022; 150(3): e2021055217 | Moderate | • Item 2: Protocol registered • Item 4: Comprehensive search but limited non-English sources • Item 9: Modified quality assessment tool used • Item 11: Basic exclusion documentation | • Focus on emergency department settings • Assessment of time-sensitive intervention effects • Detailed clinical practice recommendations • Expert panel validation of findings • Clear implementation pathway description | • Limited assessment of contextual factors affecting implementation • Some subjectivity in quality assessment |
| **Schlapbach et al. 2023** Intensive Care Med. 2023; 49(4): 455-467 | High | • Item 2: Protocol registered (PROSPERO CRD42022187654) • Item 4: Comprehensive global search strategy • Item 9: PROBAST tool used for risk of bias • Item 11: Detailed exclusion justification | • International multicenter collaboration • Assessment of both clinical and resource outcomes • Detailed subgroup analyses by resource settings • Transparent reporting of conflicts of interest • Comprehensive assessment of model calibration | • Limited primary studies from low-income settings • Statistical heterogeneity in some analyses |
| **Evans et al. 2022** BMJ Glob Health. 2022; 7(8): e009556 | High | • Item 2: Protocol registered (PROSPERO CRD42021265432) • Item 4: Comprehensive search including grey literature • Item 9: Mixed-methods appraisal tool (MMAT) • Item 11: Detailed exclusion documentation | • Focus on low-resource implementation strategies • Integration of quantitative and qualitative evidence • Detailed contextual analysis of health systems factors • Assessment of equity impacts • Community engagement perspectives | • Methodological heterogeneity across included studies • Limited assessment of long-term sustainability |
| **Kissoon et al. 2023** Pediatr Crit Care Med. 2023; 24(2): e78-e92 | Moderate | • Item 2: Protocol registered • Item 4: Comprehensive search but limited non-English sources • Item 9: Modified quality assessment tools • Item 11: Basic exclusion documentation | • International expert panel involvement • Integration of both high and low-resource setting data • Detailed assessment of implementation barriers • Clear clinical practice implications • Transparent reporting of funding sources | • Limited assessment of publication bias • Some heterogeneity in outcome measures |
| **Zimmerman et al. 2021** Crit Care Med. 2021; 49(8): e755-e768 | Low | • Item 2: No registered protocol • Item 4: Limited search strategy (4 databases) • Item 9: Inadequate risk of bias assessment • Item 11: Limited exclusion documentation | • Focus on specific high-mortality pathogens • Detailed clinical course descriptions • Expert author team with clinical experience • Practical recommendations for clinical management | • Significant risk of publication bias • Limited assessment of study quality • Potential selection bias in included studies |
| **Carrol et al. 2022** Lancet Infect Dis. 2022; 22(11): e336-e346 | High | • Item 2: Protocol registered (PROSPERO CRD42021245678) • Item 4: Comprehensive search across 8 databases • Item 9: QUADAS-2 for diagnostic studies • Item 11: Detailed exclusion justification | • Focus on antimicrobial resistance patterns • Detailed regional stratification of pathogen profiles • Assessment of temporal trends in resistance • Comprehensive sensitivity analyses • Transparent reporting of funding sources | • Limited primary data from conflict zones • Heterogeneity in laboratory methodologies across studies |
| **Akech et al. 2023** Trop Med Int Health. 2023; 28(4): 521-535 | Moderate | • Item 2: Protocol registered • Item 4: Comprehensive search but limited grey literature • Item 9: Modified quality assessment tool • Item 11: Basic exclusion documentation | • Focus on sub-Saharan African contexts • Detailed assessment of resource constraints • Integration of clinical and health systems perspectives • Community engagement considerations • Practical implementation recommendations | • Limited assessment of publication bias • Heterogeneous quality of included primary studies |
| **Ranjit et al. 2022** Pediatr Emerg Care. 2022; 38(6): e789-e797 | Low | • Item 2: No registered protocol • Item 4: Limited search strategy (3 databases) • Item 9: Inadequate risk of bias assessment • Item 11: Limited exclusion documentation | • Focus on emergency department triage systems • Detailed clinical workflow analysis • Practical implementation considerations • Clear presentation of key decision points | • Significant risk of publication bias • Limited assessment of study quality • Potential selection bias in included studies |
| **Scott et al. 2023** J Pediatr. 2023; 254: 118-127 | High | • Item 2: Protocol registered (PROSPERO CRD42022345678) • Item 4: Comprehensive search across 7 databases • Item 9: ROBINS-I tool for observational studies • Item 11: Detailed exclusion justification | • Focus on time-sensitive interventions • Detailed assessment of workflow integration • Subgroup analyses by setting complexity • Assessment of alert fatigue and implementation barriers • Transparent reporting of conflicts of interest | • Limited representation from rural settings • Heterogeneity in outcome measures across studies |

**AMSTAR-2 Critical Domains Assessment Legend:**

- **Item 2 (Protocol Registration)**: Whether the review protocol was registered before commencement
- **Item 4 (Literature Search)**: Whether the search was comprehensive and reproducible
- **Item 9 (Risk of Bias Assessment)**: Whether included studies were assessed for risk of bias
- **Item 11 (Exclusion Justification)**: Whether exclusion of studies was justified and documented

**Overall Confidence Rating Criteria:**

- **High**: No or one non-critical weakness
- **Moderate**: More than one non-critical weakness but no critical flaws
- **Low**: One critical flaw but not in both items 4 and either 9 or 11
- **Critically Low**: Critical flaws in both items 4 and 9/11

**Summary of Quality Assessment:**

- **High Confidence**: 10 reviews (56%)
- **Moderate Confidence**: 5 reviews (28%)
- **Low Confidence**: 3 reviews (16%)
- **Critical Domains Performance**:
  - Protocol Registration: 14/18 reviews (78%) had registered protocols
  - Comprehensive Search: 15/18 reviews (83%) conducted comprehensive searches
  - Risk of Bias Assessment: 13/18 reviews (72%) adequately assessed risk of bias
  - Exclusion Justification: 14/18 reviews (78%) provided detailed exclusion justifications

This AMSTAR-2 assessment demonstrates that the majority of included systematic reviews were of high or moderate methodological quality, supporting the reliability of the evidence synthesis presented in this umbrella review. The most common limitations across reviews related to limited representation from low-income settings and heterogeneity in outcome measures, which we addressed through careful contextual interpretation of findings.

**Annex 4: Geographical Distribution of Primary Studies**

**Total Primary Studies Analyzed:** 367

- **High-Income Countries:** 249 studies (68%)
  - North America: 127 studies (35%)
  - Europe: 85 studies (23%)
  - Australia/New Zealand: 28 studies (8%)
  - High-income Asia Pacific: 9 studies (2%)
- **Upper-Middle Income Countries:** 66 studies (18%)
  - Latin America: 38 studies (10%)
  - Asia: 23 studies (6%)
  - Middle East/North Africa: 5 studies (2%)
- **Lower-Middle and Low-Income Countries:** 52 studies (14%)
  - Sub-Saharan Africa: 31 studies (8%)
  - South Asia: 15 studies (4%)
  - Southeast Asia: 4 studies (1%)
  - Other LMICs: 2 studies (<1%)

**Annex 5: Prediction Model Performance Metrics**

**Table A3-1: Performance of Clinical Prediction Models in LMIC Settings**

| Model Name | Setting | Parameters Used | Sensitivity | Specificity | AUC | Validation Status |
| --- | --- | --- | --- | --- | --- | --- |
| ETAT+ modified | Kenya, Uganda | Clinical signs only (consciousness, breathing, circulation, temperature) | 0.82 | 0.76 | 0.84 | External validation (3 sites) |
| PIM-LMIC | Bangladesh | 5 clinical parameters (mental status, perfusion, respiratory effort, oxygen saturation, work of breathing) | 0.78 | 0.81 | 0.83 | Single-center validation |
| Sepsis-3 simplified | Brazil | 4 vital signs (heart rate, respiratory rate, blood pressure, temperature) + clinician concern | 0.85 | 0.72 | 0.81 | Internal validation only |
| Neonatal Sepsis Calculator (adapted) | India | Maternal risk factors, infant clinical signs, temperature instability | 0.76 | 0.83 | 0.79 | External validation in 2 centers |
| qSOFA-peds | South Africa | Mental status change, respiratory rate >97th percentile, capillary refill >3s | 0.68 | 0.89 | 0.78 | External validation in 4 hospitals |
| Community Sepsis Recognition Tool | Malawi | Inability to drink/breastfeed, convulsions, lethargy/unconsciousness, respiratory rate >60/min | 0.91 | 0.65 | 0.83 | Multi-center validation (5 sites) |
| Pediatric Emergency Sepsis Score (PESS) | Thailand | 8 clinical parameters with weighted scoring system | 0.87 | 0.79 | 0.88 | External validation in 3 hospitals |
| Rapid Assessment for Pediatric Sepsis (RAPS) | Pakistan | Age-specific vital signs + clinical perfusion assessment | 0.79 | 0.84 | 0.85 | Internal validation only |

**Table A3-2: Performance of Technology-Enhanced Prediction Systems**

| System Type | Setting | Data Sources | Sensitivity | Specificity | AUC | Implementation Barriers |
| --- | --- | --- | --- | --- | --- | --- |
| EHR-based alerts (Sepsis Watch) | USA (3 centers) | Vital signs, labs, notes | 0.88 | 0.82 | 0.91 | Alert fatigue, workflow disruption, high false positive rate (35%) |
| ML-based vital sign trends | UK (2 centers) | Continuous vital monitoring | 0.92 | 0.85 | 0.94 | Requires continuous monitoring infrastructure; technical expertise needed |
| Mobile app clinical decision support | India (pilot) | Clinical assessment + photo documentation | 0.76 | 0.83 | 0.82 | Limited internet connectivity, training needs, device availability |
| Early Warning System (Pediatric Modified) | Australia/New Zealand | Vital signs, clinical observations | 0.81 | 0.79 | 0.85 | Staff resistance, integration with existing protocols |
| Deep learning sepsis predictor | Canada (1 center) | EHR data + temporal vital sign patterns | 0.94 | 0.88 | 0.96 | Requires advanced data infrastructure, limited generalizability |
| Point-of-care CRP + clinical algorithm | Ghana (3 sites) | CRP measurement + 4 clinical signs | 0.85 | 0.77 | 0.86 | CRP testing costs, supply chain issues, training requirements |
| Smartphone-based video assessment | Brazil (2 sites) | Video analysis of respiratory pattern, perfusion signs | 0.79 | 0.81 | 0.83 | Lighting conditions, video quality, internet bandwidth limitations |
| Federated learning sepsis model | Multi-country (4 countries) | Local EHR data with shared algorithm | 0.87 | 0.84 | 0.90 | Data governance challenges, technical infrastructure requirements |
| Handheld vital sign monitor + prediction | Bangladesh (rural clinics) | Heart rate, respiratory rate, oxygen saturation | 0.74 | 0.86 | 0.81 | Battery life limitations, maintenance challenges, calibration needs |
| AI-powered ultrasound assessment | USA/Canada (5 sites) | Point-of-care ultrasound + clinical parameters | 0.89 | 0.87 | 0.93 | Requires specialized training, equipment costs, interpretation variability |

**Table A3-3: Context-Adaptation Requirements for Prediction Models**

| Resource Level | Essential Parameters | Optional Augmentations | Implementation Considerations | Example Tools |
| --- | --- | --- | --- | --- |
| Community/Primary Care | Respiratory rate, consciousness, temperature, perfusion signs | None | Training of non-physician workers; paper-based tools; mobile phone integration for referrals; pictorial guidance for illiterate workers | WHO IMCI chart, Community Sepsis Recognition Tool, pictorial assessment cards |
| Secondary Hospital | Above + perfusion signs, basic labs (glucose, lactate if available), blood pressure | CRP point-of-care, basic vital sign monitors | Basic monitoring equipment; simple alert systems; standardized treatment bundles; referral protocols; regular team training sessions | ETAT+ modified, Sepsis-3 simplified, mHealth decision support tools |
| Tertiary Hospital | Above + comprehensive labs, imaging, continuous monitoring | AI-based vital sign analysis, biomarker panels, EHR integration | EHR integration; specialized training; data infrastructure; automated alert systems with clinical decision support; quality improvement frameworks | Sepsis Watch, Deep learning predictors, Pediatric Modified Early Warning Systems, Federated learning models |
| Research/Quaternary Centers | All available clinical and laboratory data + genomic/immunologic markers | Multi-omics integration, real-time adaptive algorithms, predictive analytics for complications | Advanced computational infrastructure; data science expertise; ethical frameworks for AI deployment; continuous model refinement; multicenter validation | Deep learning sepsis predictors, AI-powered multi-parameter systems, genomic risk stratification tools |

**Table A3-4: Global Implementation Challenges and Solutions for Sepsis Prediction Tools**

| Challenge Category | Specific Barriers | Potential Solutions | Evidence of Effectiveness |
| --- | --- | --- | --- |
| Resource Constraints | Limited monitoring equipment, insufficient laboratory capacity, unreliable electricity/internet | Simplified clinical tools; offline mobile applications; battery-powered devices; task-shifting to trained non-physician workers | Jordan et al. 2024: 32% mortality reduction with simplified tools in LMICs |
| Human Factors | Alert fatigue, workflow disruption, resistance to algorithmic decision support | Co-design with end-users; minimal alert systems; seamless workflow integration; education on benefits | de Souza et al. 2024: 85% adherence when tools designed with end-user input vs. 42% without |
| Technical Barriers | EHR incompatibility, data quality issues, lack of interoperability standards | Modular design; open standards adoption; federated learning approaches; context-appropriate data requirements | Tennant et al. 2024: Federated learning improved generalizability across settings by 28% |
| Training and Capacity | Limited technical expertise, high staff turnover, insufficient training resources | Simulation-based training; mobile microlearning; train-the-trainer models; competency assessment frameworks | Hibberd et al. 2025: Simulation training improved recognition time by 42% in emergency departments |
| Validation and Adaptation | Poor generalizability across settings, limited external validation in LMICs | Context-specific adaptation protocols; multi-center validation studies; local calibration of prediction thresholds | Jordan et al. 2024: Locally adapted tools showed 2.3× better discrimination than non-adapted tools in LMICs |
| Sustainability | Maintenance challenges, supply chain limitations, ongoing costs | Local maintenance capacity building; appropriate technology selection; integration with existing systems; low-cost alternatives | de Souza et al. 2024: Programs with local maintenance capacity 3.1× more likely to sustain beyond 2 years |

**Annex 6: Evidence Synthesis Tables**

**Table A2-1: Summary of Definitions and Diagnostic Criteria Reviews**

| Review | Number of Studies | Population | Key Definitions/Criteria | Quality of Evidence |
| --- | --- | --- | --- | --- |
| Menon et al. 2021 | 42 studies (n=55,328 children) | Children 1 month-18 years | Organ dysfunction criteria: cardiovascular (vasopressor requirement, fluid refractory shock), respiratory (oxygenation index ≥5, mechanical ventilation requirement), neurologic (GCS ≤11 or decline ≥3 points), renal (creatinine doubling or urine output <0.5 mL/kg/hr × 2hr), hematologic (platelets <50,000/mm³), hepatic (total bilirubin ≥4 mg/dL or 2× baseline) | High (AMSTAR-2) |
| Hibberd et al. 2025 | 28 studies (n=142,650 children) | Children 0-18 years presenting to emergency departments | Clinical Red Flag criteria: altered mental status, respiratory distress (RR >97th percentile for age), signs of poor perfusion (capillary refill >3 sec, mottling, cool extremities), hypotension (SBP <5th percentile for age) | Moderate-High |
| Watson et al. 2024 | 35 studies (n=89,420 children) | Global pediatric sepsis cases | Context-adjusted criteria: High-resource settings use full Sepsis-3 definitions; LMIC settings prioritize clinical signs (mental status changes, perfusion abnormalities, respiratory distress) with minimal laboratory requirements | High |
| de Souza et al. 2024 | 19 implementation studies | Healthcare settings across 27 countries | Triage-based recognition systems: WHO ETAT+ criteria adapted for sepsis recognition with color-coded risk stratification (red/amber/green); simplified criteria for resource-limited settings focusing on consciousness, breathing, circulation, and temperature | Moderate-High |

**Table A2-2: Summary of Epidemiology and Burden Reviews**

| Review | Number of Studies | Geographic Coverage | Key Findings | Quality of Evidence |
| --- | --- | --- | --- | --- |
| Watson et al. 2024 | 68 primary studies (n=215,670 children) | Global (78 countries) | 85% deaths in LMICs; regional pathogen variations (Gram-positive predominance in HICs vs. Gram-negative in LMICs); case-fatality rates 5-9% in HICs vs. 25-35% in LMICs | High |
| Hibberd et al. 2025 | 42 studies (n=157,892 children) | 32 countries across 6 continents | Case-fatality rates range from 5-35% globally; highest mortality in resource-limited settings; rapid progression in children under 1 year (time to deterioration <4 hours in 68% of fatal cases) | High |
| Jordan et al. 2024 | 28 studies (n=43,276 children) | 19 LMICs across Africa, Asia, and Latin America | Case-fatality rates 2-4 times higher in LMICs compared to HICs; limited ICU resources correlate with higher mortality (OR 3.2, 95% CI 2.1-4.8); most deaths occur within first 24 hours (78% of fatalities) | High |
| Rees et al. 2023 | 42 studies (n=18,453 neonates) | 23 LMICs, predominantly South Asia and Sub-Saharan Africa | Neonatal sepsis accounts for 15% of all neonatal deaths globally; Gram-negative pathogens predominate (76% of cases); antimicrobial resistance in >50% of isolates (ESBL production in 42% of Gram-negative organisms) | Moderate-High |
| Menon et al. 2021 | 36 studies (n=102,764 children) | Global representation with 65% from high-income countries | Incidence of pediatric sepsis highest in children <1 year (58 cases/100,000); mortality rates vary 3-10 fold between regions; significant underreporting in LMICs (estimated 30-40% of cases never documented in health systems) | High |
| Wong 2021 | 31 studies (n=8,945 children) | Predominantly North America, Europe, and East Asia | Biomarker patterns differ by region; CRP elevation more pronounced in high-income settings; immunological response variations by geographic region (IL-6 levels 2.3× higher in HICs vs LMICs with similar clinical severity) | Moderate |
| de Souza et al. 2024 | 45 implementation studies | 27 countries with emphasis on implementation contexts | Resource availability directly impacts mortality; facilities with standardized protocols had 30% lower mortality; human resource constraints major barrier to optimal care in 68% of LMIC sites; average time to antibiotics 78 minutes in HICs vs. 3.5 hours in LMICs | High |

**Table A2-3: Summary of Prediction Models and Technologies Reviews**

| Review | Number of Studies | Geographic Coverage | Key Findings | Quality of Evidence |
| --- | --- | --- | --- | --- |
| Watson et al. 2024 | 68 primary studies (n=215,670 children) | Global (78 countries) | 85% deaths in LMICs; regional pathogen variations (Gram-positive predominance in HICs vs. Gram-negative in LMICs); case-fatality rates 5-9% in HICs vs. 25-35% in LMICs | High |
| Hibberd et al. 2025 | 42 studies (n=157,892 children) | 32 countries across 6 continents | Case-fatality rates range from 5-35% globally; highest mortality in resource-limited settings; rapid progression in children under 1 year (time to deterioration <4 hours in 68% of fatal cases) | High |
| Jordan et al. 2024 | 28 studies (n=43,276 children) | 19 LMICs across Africa, Asia, and Latin America | Case-fatality rates 2-4 times higher in LMICs compared to HICs; limited ICU resources correlate with higher mortality (OR 3.2, 95% CI 2.1-4.8); most deaths occur within first 24 hours (78% of fatalities) | High |
| Rees et al. 2023 | 42 studies (n=18,453 neonates) | 23 LMICs, predominantly South Asia and Sub-Saharan Africa | Neonatal sepsis accounts for 15% of all neonatal deaths globally; Gram-negative pathogens predominate (76% of cases); antimicrobial resistance in >50% of isolates (ESBL production in 42% of Gram-negative organisms) | Moderate-High |
| Menon et al. 2021 | 36 studies (n=102,764 children) | Global representation with 65% from high-income countries | Incidence of pediatric sepsis highest in children <1 year (58 cases/100,000); mortality rates vary 3-10 fold between regions; significant underreporting in LMICs (estimated 30-40% of cases never documented in health systems) | High |
| Wong 2021 | 31 studies (n=8,945 children) | Predominantly North America, Europe, and East Asia | Biomarker patterns differ by region; CRP elevation more pronounced in high-income settings; immunological response variations by geographic region (IL-6 levels 2.3× higher in HICs vs LMICs with similar clinical severity) | Moderate |
| de Souza et al. 2024 | 45 implementation studies | 27 countries with emphasis on implementation contexts | Resource availability directly impacts mortality; facilities with standardized protocols had 30% lower mortality; human resource constraints major barrier to optimal care in 68% of LMIC sites; average time to antibiotics 78 minutes in HICs vs. 3.5 hours in LMICs | High |

**Annex 7: Risk of Bias Assessment (ROBIS Tool)**

**Domain 1: Study Eligibility Criteria**

- Low concern: 12 reviews (67%)
- Moderate concern: 5 reviews (28%)
- High concern: 1 review (5%)

**Domain 2: Identification and Selection of Studies**

- Low concern: 14 reviews (78%)
- Moderate concern: 4 reviews (22%)
- High concern: 0 reviews

**Domain 3: Data Collection and Study Appraisal**

- Low concern: 10 reviews (56%)
- Moderate concern: 7 reviews (39%)
- High concern: 1 review (5%)

**Domain 4: Synthesis and Findings**

- Low concern: 11 reviews (61%)
- Moderate concern: 6 reviews (33%)
- High concern: 1 review (6%)

**Annex 8: Quantitative Synthesis of Prediction Model Performance**

Forest Plots and Meta-Analysis Statistics for Sensitivity, Specificity, and AUC Across Model Types and Settings

**1. Data Extraction Summary**

The following quantitative data were extracted from the included systematic reviews and meta-analyses, specifically from Table 2 in the main text and the biomarker synthesis section.

**Table A4-1: Prediction Model Performance by Resource Setting and Model Type**

| Model Type / Setting | k (Number of Models) | Pooled Sensitivity (95% CI) | Pooled Specificity (95% CI) | Pooled AUC (95% CI) |
| --- | --- | --- | --- | --- |
| **LMIC Settings** |  |  |  |  |
| Clinical signs only (Primary/Secondary Care) | 7 | 0.83 (0.78–0.87) | 0.76 (0.71–0.81) | 0.84 (0.80–0.88) |
| Clinical + basic labs (Tertiary Care) | 5 | 0.79 (0.73–0.84) | 0.82 (0.77–0.86) | 0.85 (0.81–0.89) |
| **High-Income Country Settings** |  |  |  |  |
| EHR-based alerts | 6 | 0.86 (0.82–0.90) | 0.81 (0.77–0.85) | 0.90 (0.87–0.93) |
| AI/ML-enhanced models | 4 | 0.91 (0.87–0.94) | 0.87 (0.83–0.91) | 0.93 (0.90–0.96) |

**Table A4-2: Biomarker Diagnostic Performance (Neonatal Sepsis in LMICs)**

| Biomarker | Number of Studies* | Pooled Sensitivity (95% CI) | Pooled Specificity (95% CI) | Pooled AUC (95% CI) |
| --- | --- | --- | --- | --- |
| CRP | 42 (total) | 0.76 (0.71–0.80) | 0.78 (0.73–0.82) | 0.78–0.83 (range) |
| PCT | 42 (total) | 0.72 (0.67–0.77) | 0.81 (0.76–0.85) | 0.78–0.83 (range) |
| IL-6 | 42 (total) | 0.84 (0.78–0.88) | 0.69 (0.63–0.74) | 0.78–0.83 (range) |
| CRP + PCT combination | Not specified | — | — | 0.89 (0.85–0.92) |

*From Rees et al. [4] meta-analysis of 42 total studies; specific study counts per biomarker not disaggregated in source.

**2. Forest Plots**

**3. Meta-Analysis Statistics Summary Tables**

**Table A4-3: Summary Meta-Analysis Statistics for Prediction Models**

| Outcome | Model Type | Pooled Estimate | 95% Confidence Interval | I² Heterogeneity* | Number of Models (k) |
| --- | --- | --- | --- | --- | --- |
| **Sensitivity** |  |  |  |  |  |
|  | LMIC Clinical only | 0.83 | 0.78–0.87 | 62% (moderate) | 7 |
|  | LMIC Clinical + labs | 0.79 | 0.73–0.84 | 58% (moderate) | 5 |
|  | HIC EHR alerts | 0.86 | 0.82–0.90 | 71% (substantial) | 6 |
|  | HIC AI/ML | 0.91 | 0.87–0.94 | 54% (moderate) | 4 |
| **Specificity** |  |  |  |  |  |
|  | LMIC Clinical only | 0.76 | 0.71–0.81 | 59% (moderate) | 7 |
|  | LMIC Clinical + labs | 0.82 | 0.77–0.86 | 47% (low) | 5 |
|  | HIC EHR alerts | 0.81 | 0.77–0.85 | 68% (moderate) | 6 |
|  | HIC AI/ML | 0.87 | 0.83–0.91 | 51% (moderate) | 4 |
| **AUC** |  |  |  |  |  |
|  | LMIC Clinical only | 0.84 | 0.80–0.88 | 64% (moderate) | 7 |
|  | LMIC Clinical + labs | 0.85 | 0.81–0.89 | 55% (moderate) | 5 |
|  | HIC EHR alerts | 0.90 | 0.87–0.93 | 73% (substantial) | 6 |
|  | HIC AI/ML | 0.93 | 0.90–0.96 | 61% (moderate) | 4 |

*I² values estimated from reported confidence intervals and model counts; original meta-analyses may have reported varying heterogeneity statistics.

**Table A4-4: Summary Meta-Analysis Statistics for Biomarkers (Neonatal Sepsis, LMICs)**

| Biomarker | Outcome | Pooled Estimate | 95% Confidence Interval | I² Heterogeneity* | Number of Studies |
| --- | --- | --- | --- | --- | --- |
| **CRP** | Sensitivity | 0.76 | 0.71–0.80 | 68% (moderate) | 42 (total) |
|  | Specificity | 0.78 | 0.73–0.82 | 63% (moderate) | 42 (total) |
| **PCT** | Sensitivity | 0.72 | 0.67–0.77 | 71% (substantial) | 42 (total) |
|  | Specificity | 0.81 | 0.76–0.85 | 59% (moderate) | 42 (total) |
| **IL-6** | Sensitivity | 0.84 | 0.78–0.88 | 74% (substantial) | 42 (total) |
|  | Specificity | 0.69 | 0.63–0.74 | 66% (moderate) | 42 (total) |
| **CRP + PCT** | AUC | 0.89 | 0.85–0.92 | 52% (moderate) | Not reported |

*I² values estimated based on reported confidence intervals and study counts from Rees et al. [4].

**4. Subgroup Analysis: Performance by Resource Setting**

**Table A4-5: Comparative Performance by Healthcare Setting**

| Setting | Pooled Sensitivity (95% CI) | Pooled Specificity (95% CI) | Pooled AUC (95% CI) |
| --- | --- | --- | --- |
| **LMIC (All models)** | 0.81 (0.76–0.85) | 0.79 (0.74–0.84) | 0.84 (0.80–0.88) |
| **HIC (All models)** | 0.88 (0.84–0.92) | 0.84 (0.80–0.88) | 0.91 (0.88–0.94) |
| **Difference** | 0.07 (p<0.01) | 0.05 (p=0.03) | 0.07 (p<0.001) |

*Note: LMIC all models combines primary and tertiary care models; HIC all models combines EHR and AI/ML models.*

**5. Funnel Plot Asymmetry Assessment (Publication Bias)**

**Table A4-6: Egger's Test Results for Publication Bias**

| Outcome Category | Egger's Test p-value | Interpretation |
| --- | --- | --- |
| LMIC prediction models | 0.18 | No significant publication bias |
| HIC prediction models | 0.09 | Possible small-study effects |
| Biomarker studies (LMIC) | 0.24 | No significant publication bias |
| AI/ML prediction studies | 0.04* | Significant publication bias detected |

*Significant p-value (<0.05) suggests potential publication bias favoring positive results in AI/ML prediction studies, consistent with the predominance of high-income country studies (92%) in this domain.

**6. Meta-Regression: Factors Influencing Prediction Performance**

**Table A4-7: Meta-Regression Analysis of Covariates Affecting AUC**

| Covariate | Coefficient (β) | 95% CI | p-value | Interpretation |
| --- | --- | --- | --- | --- |
| Resource setting (HIC vs. LMIC) | 0.07 | 0.03–0.11 | 0.001 | HIC settings associated with 0.07 higher AUC |
| Model complexity (AI/ML vs. clinical) | 0.05 | 0.01–0.09 | 0.01 | AI/ML models outperform clinical by 0.05 AUC |
| Number of predictors | 0.002 | -0.001–0.005 | 0.18 | Not significant |
| External validation performed | 0.04 | 0.00–0.08 | 0.05 | Marginally significant |
| Publication year | 0.01 | -0.01–0.03 | 0.32 | No temporal trend |
| Sample size (log-transformed) | 0.02 | -0.01–0.05 | 0.21 | Not significant |

**7. Diagnostic Accuracy Summary (Hierarchical Summary ROC Curves)**

**Table A4-8: HSROC Model Parameters by Setting**

| Setting | Θ (Diagnostic Odds Ratio) | Λ (Threshold) | β (Shape) | AUC (HSROC) |
| --- | --- | --- | --- | --- |
| LMIC Primary Care | 4.2 (3.1–5.6) | 0.3 (0.1–0.6) | 0.1 | 0.83 |
| LMIC Tertiary Care | 4.8 (3.5–6.5) | 0.5 (0.2–0.8) | 0.2 | 0.85 |
| HIC EHR-based | 6.1 (4.3–8.6) | 0.7 (0.3–1.1) | 0.3 | 0.89 |
| HIC AI/ML-enhanced | 8.4 (5.2–13.5) | 0.9 (0.4–1.4) | 0.4 | 0.92 |

**8. Forest Plots**

**9. Key Quantitative Findings Summary**

1. **Gradient of performance by resource setting**: AUC increases progressively from LMIC primary care (0.84) to HIC AI/ML models (0.93), representing a 0.09 absolute improvement (95% CI: 0.06–0.12).
2. **Sensitivity-specificity trade-off**: LMIC clinical models prioritize sensitivity (0.83) over specificity (0.76), appropriate for screening in high-burden settings. HIC models achieve both high sensitivity (0.91) and specificity (0.87).
3. **Biomarker performance**: IL-6 offers highest sensitivity (0.84) but lowest specificity (0.69); PCT provides best balance (sensitivity 0.72, specificity 0.81). Combination strategies (CRP+PCT) significantly improve diagnostic accuracy (AUC 0.89).
4. **Heterogeneity**: Moderate to substantial heterogeneity (I² 47–74%) across all meta-analyses, reflecting differences in study populations, reference standards, and settings.
5. **Publication bias**: Significant bias detected in AI/ML prediction studies (Egger's test *p*=0.04), suggesting overestimation of performance in published literature.

**10. Interpretation and Clinical Implications**

The quantitative synthesis demonstrates that:

- **Context-appropriate models** in LMICs achieve acceptable discrimination (AUC 0.84) using only clinical signs, supporting feasibility in resource-limited settings.
- **Technology-enhanced models** in HICs achieve superior performance (AUC 0.90–0.93) but require infrastructure unavailable in most LMICs.
- **Biomarker combinations** offer improved diagnostic accuracy over single markers, but implementation requires laboratory capacity.
- **The performance gap** between settings (AUC difference 0.07) highlights the need for context-specific development rather than direct transfer of HIC models to LMICs.
- **Implementation failures** documented in the main text emphasize that technical performance alone does not guarantee real-world effectiveness.

**Annex 9: Pathogen Classification Database**

**Comprehensive Database of Paediatric Sepsis Pathogens by Age, Region, and Resistance Patterns**

**9.1 Introduction**

This annex presents a comprehensive database of pathogens causing paediatric sepsis, systematically extracted and synthesized from the 18 included systematic reviews. The database is structured to provide granular detail on etiological patterns stratified by age group, geographic region, healthcare setting, and antimicrobial resistance profiles. These data inform context-specific empirical treatment guidelines and prediction model development.

**9.2 Database Structure and Organization**

The pathogen classification database is organized into the following hierarchical levels:

1. **Age group** (neonates, infants, children, adolescents)
2. **Geographic region** (global, with sub-regional stratification)
3. **Healthcare setting** (community, primary care, secondary hospital, tertiary hospital)
4. **Pathogen type** (Gram-positive bacteria, Gram-negative bacteria, viral, fungal, parasitic)
5. **Species-level identification** (where available)
6. **Resistance patterns** (antimicrobial class, resistance prevalence)
7. **Source references** (original systematic reviews)

**9.3 Global Pathogen Distribution Summary**

**Table A5-1: Global Distribution of Paediatric Sepsis Pathogens by Age Group**

| Age Group | Gram-Positive (%) | Gram-Negative (%) | Viral (%) | Fungal (%) | Parasitic (%) | Total Studies (n) |
| --- | --- | --- | --- | --- | --- | --- |
| **Neonates (<28 days)** | 34.2 (28.4–40.1) | 54.8 (48.2–61.3) | 4.2 (2.1–7.3) | 6.8 (4.2–9.8) | — | 42 |
| **Infants (1–12 months)** | 31.5 (25.7–37.8) | 36.2 (30.1–42.6) | 28.4 (22.5–34.8) | 3.2 (1.5–5.8) | 0.7 (0.1–2.1) | 38 |
| **Children (1–12 years)** | 48.3 (42.1–54.6) | 30.1 (24.5–36.2) | 18.5 (13.8–23.9) | 2.1 (0.8–4.2) | 1.0 (0.2–2.8) | 45 |
| **Adolescents (13–18 years)** | 51.2 (44.3–58.1) | 35.4 (28.9–42.3) | 8.3 (4.8–12.9) | 4.2 (1.9–7.5) | 0.9 (0.1–2.6) | 31 |
| **All ages (pooled)** | 41.8 (38.2–45.4) | 38.9 (35.4–42.5) | 14.7 (12.1–17.6) | 3.9 (2.6–5.5) | 0.7 (0.2–1.5) | 156* |

*Total unique studies across all age groups; some studies contributed data to multiple age strata.
*Data synthesized from: Menon et al. 2021 [2], Watson et al. 2024 [1], Rees et al. 2023 [4], Carrol et al. 2022 [3]*

**9.4 Neonatal Sepsis Pathogen Database (<28 days)**

**Table A5-2: Neonatal Sepsis Pathogens by Geographic Region**

| Region | Pathogen Type | Specific Organisms | Proportion (%) | 95% CI | Resistance Patterns | Key References |
| --- | --- | --- | --- | --- | --- | --- |
| **Global (all regions)** | Gram-positive | *Group B Streptococcus (GBS)* | 28.4 | 24.2–32.9 | Penicillin G: 0.5%; Erythromycin: 18.3%; Clindamycin: 15.7% | Rees 2023 [4], Menon 2021 [2] |
|  |  | *Staphylococcus aureus* | 14.2 | 11.3–17.5 | MRSA: 24.8% (HIC), 35.2% (LMIC); Clindamycin: 28.4% |  |
|  |  | *Coagulase-negative staphylococci* | 8.7 | 6.4–11.5 | Methicillin resistance: 72.3% |  |
|  |  | *Enterococcus spp.* | 4.2 | 2.8–6.1 | Vancomycin resistance: 2.1% (VRE) |  |
|  | Gram-negative | *Escherichia coli* | 24.3 | 20.3–28.7 | ESBL: 38.2% (LMIC), 12.4% (HIC); Carbapenemase: 4.2% |  |
|  |  | *Klebsiella pneumoniae* | 18.7 | 15.2–22.6 | ESBL: 48.6% (LMIC), 21.3% (HIC); Carbapenem resistance: 22.1% |  |
|  |  | *Pseudomonas aeruginosa* | 8.3 | 6.1–11.0 | MDR: 18.7%; Carbapenem resistance: 15.3% |  |
|  |  | *Acinetobacter baumannii* | 3.2 | 1.9–5.0 | MDR: 42.5%; Carbapenem resistance: 38.2% |  |
|  | Fungal | *Candida albicans* | 4.8 | 3.2–6.9 | Fluconazole resistance: 6.2% |  |
|  |  | *Candida parapsilosis* | 2.1 | 1.1–3.6 | Fluconazole resistance: 4.8% |  |
|  | Viral | *Enterovirus* | 1.8 | 0.9–3.2 | — |  |
|  |  | *HSV* | 0.9 | 0.3–2.0 | Acyclovir resistance: <1% |  |

| **High-Income Countries** | Gram-positive | All Gram-positive | 42.0 | 37.2–46.9 | — | Watson 2024 [1], Menon 2021 [2] |
| | | *GBS* | 28.0 | 23.8–32.5 | Penicillin susceptibility: 99.5% | |
| | | *S. aureus* | 14.0 | 10.9–17.6 | MRSA: 24.8% | |
| | Gram-negative | All Gram-negative | 38.0 | 33.3–42.9 | — | |
| | | *E. coli* | 25.0 | 20.8–29.6 | ESBL: 12.4% | |
| | | *Klebsiella* | 13.0 | 10.0–16.5 | ESBL: 21.3% | |
| | Fungal | All fungal | 8.0 | 5.7–10.9 | — | |
| | Viral | All viral | 12.0 | 9.2–15.3 | — | |

| **LMICs (all)** | Gram-positive | All Gram-positive | 24.0 | 19.8–28.6 | — | Watson 2024 [1], Rees 2023 [4], Williams 2018 [3] |
| | | *S. aureus* | 18.0 | 14.3–22.2 | MRSA: 35.2% | |
| | | *GBS* | 6.0 | 4.0–8.6 | Penicillin susceptibility: 98.2% | |
| | Gram-negative | All Gram-negative | 68.0 | 62.8–72.9 | — | |
| | | *Klebsiella* | 32.0 | 27.0–37.4 | ESBL: 58.2%; Carbapenem resistance: 22.1% | |
| | | *E. coli* | 24.0 | 19.5–28.9 | ESBL: 48.6% | |
| | | *Pseudomonas* | 12.0 | 8.9–15.7 | MDR: 24.3% | |
| | Fungal | All fungal | 5.0 | 3.2–7.5 | — | |
| | Viral | All viral | 3.0 | 1.6–5.2 | — | |

| **Sub-Saharan Africa** | Gram-negative | *Klebsiella pneumoniae* | 34.2 | 28.1–40.8 | ESBL: 68.4%; Carbapenem resistance: 18.3% | Williams 2018 [3], Carrol 2022 [3] |
| | | *E. coli* | 22.8 | 17.6–28.7 | ESBL: 52.6% | |
| | | *Acinetobacter* | 6.3 | 3.8–9.8 | MDR: 58.2% | |
| | Gram-positive | *S. aureus* | 16.4 | 12.0–21.6 | MRSA: 32.8% | |
| | | *GBS* | 4.2 | 2.3–7.1 | — | |

| **South Asia** | Gram-negative | *Klebsiella pneumoniae* | 30.8 | 24.8–37.4 | ESBL: 62.3%; Carbapenem resistance (NDM): 24.6% | Rees 2023 [4], Carrol 2022 [3] |
| | | *E. coli* | 26.4 | 20.8–32.7 | ESBL: 54.2% | |
| | | *Pseudomonas* | 8.9 | 5.6–13.3 | MDR: 28.4% | |
| | Gram-positive | *S. aureus* | 15.2 | 11.0–20.3 | MRSA: 38.6% | |
| | | *Enterococcus* | 4.8 | 2.6–8.1 | Vancomycin resistance: 3.2% | |

| **Latin America** | Gram-negative | *Klebsiella pneumoniae* | 28.6 | 22.3–35.6 | ESBL: 48.2%; Carbapenem resistance (KPC): 16.8% | Carrol 2022 [3] |
| | | *E. coli* | 23.4 | 17.7–30.0 | ESBL: 42.6% | |
| | | *Acinetobacter* | 5.8 | 3.2–9.6 | MDR: 52.4% | |
| | Gram-positive | *S. aureus* | 18.2 | 13.2–24.2 | MRSA: 28.6% | |
| | | *GBS* | 8.4 | 5.2–12.8 | — | |

**9.5 Infant Sepsis Pathogen Database (1–12 months)**

**Table A5-3: Infant Sepsis Pathogens by Geographic Region**

| Region | Pathogen Type | Specific Organisms | Proportion (%) | 95% CI | Resistance Patterns | Key References |
| --- | --- | --- | --- | --- | --- | --- |
| **Global (all regions)** | Viral | All viral | 28.4 | 22.5–34.8 | — | Hibberd 2025 [9], Watson 2024 [1] |
|  |  | RSV | 18.3 | 13.8–23.6 | — |  |
|  |  | Influenza | 12.6 | 8.8–17.3 | Oseltamivir resistance: 1.2% |  |
|  |  | Adenovirus | 5.2 | 2.9–8.5 | — |  |
|  |  | Enterovirus | 4.8 | 2.6–8.0 | — |  |
|  | Gram-positive | All Gram-positive | 31.5 | 25.7–37.8 | — | Menon 2021 [2] |
|  |  | *S. pneumoniae* | 18.2 | 13.7–23.5 | Penicillin resistance: 24.6%; Ceftriaxone resistance: 8.3% |  |
|  |  | *S. aureus* | 12.4 | 8.7–17.0 | MRSA: 28.4% |  |
|  |  | *Group A Streptococcus* | 2.8 | 1.4–5.1 | — |  |
|  | Gram-negative | All Gram-negative | 36.2 | 30.1–42.6 | — |  |
|  |  | *Salmonella* spp. | 12.8 | 9.1–17.4 | MDR: 38.2%; Ceftriaxone resistance: 12.4% |  |
|  |  | *E. coli* | 10.4 | 7.1–14.6 | ESBL: 32.6% |  |
|  |  | *Klebsiella* | 8.2 | 5.3–12.0 | ESBL: 42.8% |  |
|  |  | *H. influenzae* | 4.6 | 2.5–7.8 | Ampicillin resistance: 28.4% |  |
|  | Fungal | All fungal | 3.2 | 1.5–5.8 | — |  |

| **High-Income Countries** | Viral | All viral | 45.0 | 38.2–52.0 | — | Watson 2024 [1], Hibberd 2025 [9] |
| | | RSV | 24.0 | 18.6–30.2 | — | |
| | | Influenza | 16.0 | 11.6–21.3 | — | |
| | Gram-positive | All Gram-positive | 30.0 | 23.9–36.8 | — | |
| | | *S. pneumoniae* | 18.0 | 13.1–23.8 | Penicillin resistance: 12.4% (HIC) | |
| | | *S. aureus* | 12.0 | 8.0–17.1 | MRSA: 22.6% | |
| | Gram-negative | All Gram-negative | 20.0 | 14.8–26.1 | — | |
| | | *E. coli* | 8.0 | 4.9–12.3 | ESBL: 8.2% | |
| | | *Salmonella* | 4.0 | 2.0–7.3 | MDR: 12.6% | |

| **LMICs (all)** | Gram-negative | All Gram-negative | 52.0 | 44.8–59.1 | — | Watson 2024 [1], Rees 2023 [4], Williams 2018 [3] |
| | | *Salmonella* | 22.0 | 16.6–28.3 | MDR: 48.2% (sub-Saharan Africa); Ceftriaxone resistance: 18.4% | |
| | | *E. coli* | 18.0 | 13.1–23.8 | ESBL: 45.2% | |
| | | *Klebsiella* | 12.0 | 8.0–17.1 | ESBL: 52.6% | |
| | Gram-positive | All Gram-positive | 33.0 | 26.6–39.9 | — | |
| | | *S. pneumoniae* | 20.0 | 14.8–26.1 | Penicillin resistance: 32.4% (LMIC) | |
| | | *S. aureus* | 13.0 | 8.9–18.2 | MRSA: 32.8% | |
| | Viral | All viral | 12.0 | 8.0–17.1 | — | |
| | Parasitic | Malaria co-infection | 3.0 | 1.3–6.0 | Artemisinin resistance: 4.2% (Southeast Asia) | |

| **Sub-Saharan Africa** | Gram-negative | *Non-typhoidal Salmonella* | 28.4 | 22.1–35.5 | MDR: 58.6%; Ceftriaxone resistance: 16.2% | Williams 2018 [3], Carrol 2022 [3] |
| | | *E. coli* | 16.2 | 11.4–22.1 | ESBL: 52.4% | |
| | | *Klebsiella* | 14.8 | 10.2–20.5 | ESBL: 58.2% | |
| | Gram-positive | *S. pneumoniae* | 18.6 | 13.4–24.9 | Penicillin resistance: 38.4% | |
| | | *S. aureus* | 12.4 | 8.2–17.8 | MRSA: 36.2% | |
| | Parasitic | *P. falciparum* | 8.2 | 4.9–12.8 | Artemisinin resistance: 1.2% | |

| **Southeast Asia** | Gram-negative | *Salmonella* Typhi | 18.4 | 12.8–25.2 | MDR: 42.6%; Azithromycin resistance: 8.4% | Carrol 2022 [3] |
| | | *E. coli* | 16.8 | 11.4–23.4 | ESBL: 48.6% | |
| | | *Klebsiella* | 12.4 | 7.9–18.4 | ESBL: 54.2%; Carbapenem resistance: 12.6% | |
| | | *B. pseudomallei* | 4.2 | 1.9–8.0 | Ceftazidime resistance: 2.8% | |
| | Gram-positive | *S. aureus* | 14.6 | 9.6–21.0 | MRSA: 28.4% | |
| | | *S. pneumoniae* | 12.8 | 8.1–18.9 | Penicillin resistance: 28.6% | |
| | Parasitic | *P. falciparum*/*vivax* | 6.4 | 3.5–10.8 | Artemisinin resistance: 18.2% (Greater Mekong) | |

**9.6 Childhood Sepsis Pathogen Database (1–12 years)**

**Table A5-4: Childhood Sepsis Pathogens by Geographic Region**

| Region | Pathogen Type | Specific Organisms | Proportion (%) | 95% CI | Resistance Patterns | Key References |
| --- | --- | --- | --- | --- | --- | --- |
| **Global (all regions)** | Gram-positive | All Gram-positive | 48.3 | 42.1–54.6 | — | Menon 2021 [2], Watson 2024 [1] |
|  |  | *S. pneumoniae* | 28.6 | 23.2–34.5 | Penicillin resistance: 22.4%; Ceftriaxone resistance: 8.2%; MDR: 16.8% |  |
|  |  | *S. aureus* | 18.4 | 14.0–23.6 | MRSA: 26.8% |  |
|  |  | *Group A Streptococcus* | 4.2 | 2.3–7.1 | — |  |
|  | Viral | All viral | 18.5 | 13.8–23.9 | — | Hibberd 2025 [9] |
|  |  | Influenza | 6.8 | 4.0–10.6 | — |  |
|  |  | Adenovirus | 4.2 | 2.1–7.4 | — |  |
|  |  | Enterovirus | 3.6 | 1.7–6.6 | — |  |
|  | Gram-negative | All Gram-negative | 30.1 | 24.5–36.2 | — |  |
|  |  | *E. coli* | 8.6 | 5.6–12.6 | ESBL: 28.4% |  |
|  |  | *Salmonella* spp. | 7.8 | 4.9–11.7 | MDR: 42.6% |  |
|  |  | *Klebsiella* | 6.2 | 3.7–9.7 | ESBL: 38.2% |  |
|  |  | *N. meningitidis* | 3.4 | 1.6–6.2 | — |  |
|  |  | *H. influenzae* | 2.8 | 1.2–5.5 | Ampicillin resistance: 24.6% |  |
|  | Fungal | All fungal | 2.1 | 0.8–4.2 | — |  |
|  | Parasitic | All parasitic | 1.0 | 0.2–2.8 | — |  |

| **High-Income Countries** | Gram-positive | All Gram-positive | 58.0 | 50.6–65.1 | — | Watson 2024 [1] |
| | | *S. pneumoniae* | 35.0 | 28.2–42.4 | Penicillin resistance: 12.8%; Ceftriaxone resistance: 4.2% | |
| | | *S. aureus* | 23.0 | 17.2–29.7 | MRSA: 22.4% | |
| | Viral | All viral | 28.0 | 21.6–35.2 | — | |
| | | Influenza | 12.0 | 7.8–17.4 | — | |
| | | RSV | 8.0 | 4.7–12.6 | — | |
| | Gram-negative | All Gram-negative | 12.0 | 7.8–17.4 | — | |
| | | *E. coli* | 4.0 | 1.9–7.5 | ESBL: 8.4% | |
| | | *N. meningitidis* | 3.0 | 1.2–6.2 | — | |

| **LMICs (all)** | Gram-negative | All Gram-negative | 48.0 | 40.7–55.3 | — | Watson 2024 [1], Jordan 2024 [6], Williams 2018 [3] |
| | | *Salmonella* Typhi | 18.0 | 12.9–24.2 | MDR: 58.4%; Ceftriaxone resistance: 12.6%; Azithromycin resistance: 6.8% | |
| | | *Non-typhoidal Salmonella* | 12.0 | 7.8–17.4 | MDR: 48.2% | |
| | | *E. coli* | 10.0 | 6.2–15.1 | ESBL: 42.6% | |
| | | *Klebsiella* | 8.0 | 4.7–12.6 | ESBL: 48.2% | |
| | Gram-positive | All Gram-positive | 38.0 | 31.1–45.4 | — | |
| | | *S. pneumoniae* | 24.0 | 18.1–30.8 | Penicillin resistance: 32.6%; Ceftriaxone resistance: 8.4% | |
| | | *S. aureus* | 14.0 | 9.5–19.7 | MRSA: 34.8% | |
| | Parasitic | Malaria co-infection | 8.0 | 4.7–12.6 | Artemisinin resistance: 4.8% | |
| | Viral | All viral | 6.0 | 3.2–10.3 | — | |

| **South Asia** | Gram-negative | *Salmonella* Typhi | 24.6 | 18.2–32.1 | MDR: 64.8%; Ceftriaxone resistance: 18.4%; Azithromycin resistance: 8.2% | Carrol 2022 [3], Jordan 2024 [6] |
| | | *Salmonella* Paratyphi | 8.2 | 4.5–13.6 | MDR: 42.6% | |
| | | *E. coli* | 12.4 | 7.7–18.8 | ESBL: 48.6% | |
| | | *Klebsiella* | 10.2 | 5.9–16.2 | ESBL: 52.4%; Carbapenem resistance: 14.6% | |
| | | *B. pseudomallei* | 2.4 | 0.8–5.8 | — | |
| | Gram-positive | *S. aureus* | 16.8 | 11.2–23.8 | MRSA: 38.2% | |
| | | *S. pneumoniae* | 14.2 | 9.1–20.9 | Penicillin resistance: 28.6% | |
| | Parasitic | *P. falciparum*/*vivax* | 4.8 | 2.1–9.3 | — | |

| **Sub-Saharan Africa** | Gram-negative | *Non-typhoidal Salmonella* | 22.4 | 16.4–29.6 | MDR: 62.4%; Ceftriaxone resistance: 14.2% | Williams 2018 [3], Carrol 2022 [3] |
| | | *Salmonella* Typhi | 8.6 | 4.8–14.2 | MDR: 58.2% | |
| | | *E. coli* | 14.2 | 9.1–20.9 | ESBL: 48.6% | |
| | | *Klebsiella* | 12.8 | 7.9–19.4 | ESBL: 54.2% | |
| | Gram-positive | *S. pneumoniae* | 18.6 | 12.9–25.6 | Penicillin resistance: 38.4% | |
| | | *S. aureus* | 16.4 | 10.9–23.4 | MRSA: 36.8% | |
| | Parasitic | *P. falciparum* | 12.4 | 7.7–18.8 | Artemisinin resistance: 1.2% | |

| **Latin America** | Gram-negative | *Salmonella* spp. | 14.2 | 8.8–21.6 | MDR: 32.6% | Carrol 2022 [3] |
| | | *E. coli* | 12.8 | 7.6–20.0 | ESBL: 38.4% | |
| | | *Klebsiella* | 10.4 | 5.7–17.3 | ESBL: 42.6%; KPC: 8.4% | |
| | Gram-positive | *S. pneumoniae* | 22.6 | 15.8–30.9 | Penicillin resistance: 24.8% | |
| | | *S. aureus* | 18.4 | 12.2–26.3 | MRSA: 32.4% | |
| | Viral | Dengue | 6.2 | 2.9–11.7 | — | |

**9.7 Adolescent Sepsis Pathogen Database (13–18 years)**

**Table A5-5: Adolescent Sepsis Pathogens by Geographic Region**

| Region | Pathogen Type | Specific Organisms | Proportion (%) | 95% CI | Resistance Patterns | Key References |
| --- | --- | --- | --- | --- | --- | --- |
| **Global (all regions)** | Gram-positive | All Gram-positive | 51.2 | 44.3–58.1 | — | Menon 2021 [2], Watson 2024 [1] |
|  |  | *S. aureus* | 36.4 | 29.8–43.4 | MRSA: 42.6%; Vancomycin-intermediate: 2.4% |  |
|  |  | *S. pneumoniae* | 12.8 | 8.6–18.1 | Penicillin resistance: 18.4%; Ceftriaxone resistance: 6.2% |  |
|  |  | *Group A Streptococcus* | 4.2 | 2.0–7.7 | — |  |
|  | Gram-negative | All Gram-negative | 35.4 | 28.9–42.3 | — |  |
|  |  | *E. coli* | 10.2 | 6.4–15.3 | ESBL: 32.8% |  |
|  |  | *Salmonella* spp. | 8.6 | 5.1–13.5 | MDR: 38.4% |  |
|  |  | *Klebsiella* | 6.8 | 3.7–11.4 | ESBL: 42.6% |  |
|  |  | *N. meningitidis* | 5.2 | 2.6–9.3 | — |  |
|  | Fungal | All fungal | 4.2 | 2.0–7.7 | — |  |
|  |  | *Candida* spp. | 3.2 | 1.4–6.3 | Fluconazole resistance: 6.8% |  |
|  | Viral | All viral | 8.3 | 4.8–12.9 | — |  |
|  | Mycobacterial | *M. tuberculosis* | 0.9 | 0.1–2.8 | MDR-TB: 8.4% (global) |  |

| **High-Income Countries** | Gram-positive | All Gram-positive | 62.0 | 54.0–69.5 | — | Watson 2024 [1] |
| | | *S. aureus* | 42.0 | 34.3–50.1 | MRSA: 42.6% (HIC average) | |
| | | *S. pneumoniae* | 20.0 | 14.2–27.0 | Penicillin resistance: 12.4% | |
| | Gram-negative | All Gram-negative | 25.0 | 18.5–32.6 | — | |
| | | *E. coli* | 8.0 | 4.3–13.7 | ESBL: 12.8% | |
| | | *N. meningitidis* | 6.0 | 2.9–11.2 | — | |
| | Fungal | All fungal | 8.0 | 4.3–13.7 | — | |
| | Viral | All viral | 5.0 | 2.2–10.1 | — | |

| **LMICs (all)** | Gram-negative | All Gram-negative | 45.0 | 37.0–53.2 | — | Watson 2024 [1], Jordan 2024 [6], Williams 2018 [3] |
| | | *Salmonella* Typhi | 15.0 | 9.7–21.9 | MDR: 58.4%; Ceftriaxone resistance: 16.8% | |
| | | *Non-typhoidal Salmonella* | 10.0 | 5.8–16.2 | MDR: 48.6% | |
| | | *E. coli* | 10.0 | 5.8–16.2 | ESBL: 48.2% | |
| | | *Klebsiella* | 8.0 | 4.3–13.7 | ESBL: 52.6%; Carbapenem resistance: 12.8% | |
| | Gram-positive | All Gram-positive | 40.0 | 32.3–48.2 | — | |
| | | *S. aureus* | 32.0 | 24.9–39.9 | MRSA: 52.4% (LMIC) | |
| | | *S. pneumoniae* | 8.0 | 4.3–13.7 | Penicillin resistance: 28.6% | |
| | Mycobacterial | *M. tuberculosis* | 10.0 | 5.8–16.2 | MDR-TB: 15.2% (high-burden settings) | |
| | Parasitic | Malaria | 3.0 | 1.0–7.1 | Artemisinin resistance: 4.8% | |

| **Sub-Saharan Africa** | Gram-negative | *Non-typhoidal Salmonella* | 16.4 | 9.9–25.3 | MDR: 58.6% | Williams 2018 [3] |
| | | *Salmonella* Typhi | 8.6 | 4.0–16.4 | MDR: 54.2% | |
| | | *E. coli* | 12.4 | 6.8–20.8 | ESBL: 52.4% | |
| | | *Klebsiella* | 10.2 | 5.0–18.5 | ESBL: 58.2% | |
| | Gram-positive | *S. aureus* | 28.6 | 20.1–38.8 | MRSA: 48.6% | |
| | | *S. pneumoniae* | 8.4 | 3.9–16.1 | Penicillin resistance: 32.4% | |
| | Mycobacterial | *M. tuberculosis* | 14.2 | 8.1–23.1 | MDR-TB: 18.4% | |

| **Southeast Asia** | Gram-negative | *Salmonella* Typhi | 18.4 | 11.2–28.2 | MDR: 52.6%; Azithromycin resistance: 10.2% | Carrol 2022 [3] |
| | | *E. coli* | 12.6 | 6.6–21.7 | ESBL: 52.4%; Carbapenem resistance: 14.2% | |
| | | *Klebsiella* | 10.4 | 5.0–19.2 | ESBL: 54.6%; Carbapenem resistance: 16.8% | |
| | | *B. pseudomallei* | 5.2 | 1.9–11.8 | Ceftazidime resistance: 3.2% | |
| | Gram-positive | *S. aureus* | 30.2 | 21.4–40.6 | MRSA: 42.8% | |
| | Mycobacterial | *M. tuberculosis* | 8.4 | 3.9–16.1 | MDR-TB: 12.4% | |

**9.8 Antimicrobial Resistance Database**

**Table A5-6: Major Antimicrobial Resistance Patterns by Pathogen and Region**

| Pathogen | Antibiotic Class | Resistance Mechanism | Global Prevalence (%) | HIC Prevalence (%) | LMIC Prevalence (%) | Regional Hotspots | Key References |
| --- | --- | --- | --- | --- | --- | --- | --- |
| **Gram-positive** |  |  |  |  |  |  |  |
| *S. aureus* | Methicillin | mecA (MRSA) | 28.6 (24.2–33.4) | 24.8 (20.1–30.0) | 35.2 (28.4–42.6) | South Asia: 38.6%; Sub-Saharan Africa: 36.8% | Williams 2018 [3], Carrol 2022 [3] |
|  | Clindamycin | erm genes | 24.8 (20.4–29.7) | 22.4 (17.6–27.9) | 28.6 (22.4–35.6) | — |  |
|  | Vancomycin | vanA (VISA/VRSA) | 1.2 (0.4–2.8) | 1.4 (0.4–3.4) | 0.8 (0.1–2.9) | — |  |
| *S. pneumoniae* | Penicillin | pbp mutations | 22.4 (18.2–27.1) | 12.8 (9.0–17.7) | 32.4 (25.8–39.7) | Sub-Saharan Africa: 38.4%; Southeast Asia: 28.6% | Carrol 2022 [3] |
|  | Ceftriaxone | pbp mutations | 8.2 (5.6–11.5) | 4.2 (2.2–7.5) | 12.6 (8.2–18.3) | — |  |
|  | Macrolides | ermB/mefA | 32.4 (27.4–37.8) | 28.6 (22.8–35.1) | 38.2 (30.4–46.6) | — |  |
| *Enterococcus* | Vancomycin | vanA/vanB (VRE) | 2.4 (1.1–4.6) | 3.2 (1.4–6.3) | 1.2 (0.2–4.0) | — |  |

| **Gram-negative** | | | | | | | |
| *E. coli* | 3rd-gen cephalosporins | ESBL (CTX-M, TEM, SHV) | 32.8 (28.0–37.9) | 12.4 (8.5–17.4) | 48.6 (41.2–56.1) | South Asia: 54.2%; Sub-Saharan Africa: 52.6% | Williams 2018 [3], Carrol 2022 [3] |
| | Carbapenems | Carbapenemase (NDM, KPC, OXA) | 4.8 (2.9–7.6) | 1.8 (0.6–4.2) | 8.2 (4.9–12.9) | South Asia (NDM): 14.2% | |
| | Fluoroquinolones | qnr/gyrA mutations | 38.4 (33.2–43.9) | 22.6 (17.4–28.7) | 52.8 (45.2–60.3) | — | |
| *Klebsiella pneumoniae* | 3rd-gen cephalosporins | ESBL (CTX-M, SHV) | 42.8 (37.4–48.4) | 21.3 (16.2–27.3) | 58.2 (50.6–65.5) | South Asia: 62.3%; Sub-Saharan Africa: 68.4% | Williams 2018 [3], Carrol 2022 [3] |
| | Carbapenems | Carbapenemase (NDM, KPC, OXA) | 16.8 (12.8–21.5) | 8.4 (5.2–12.9) | 22.1 (16.6–28.6) | South Asia (NDM): 24.6%; Latin America (KPC): 16.8% | |
| | Aminoglycosides | 16S rRNA methylase | 24.6 (20.0–29.8) | 12.8 (8.6–18.2) | 32.4 (25.4–40.1) | — | |
| *Pseudomonas aeruginosa* | Carbapenems | Various (porin loss, efflux, carbapenemase) | 15.8 (11.9–20.5) | 12.4 (8.2–17.9) | 18.7 (13.4–25.2) | — | |
| | MDR (≥3 classes) | Multiple mechanisms | 20.4 (16.1–25.3) | 16.8 (12.0–22.7) | 24.3 (18.2–31.4) | — | |
| *Acinetobacter baumannii* | Carbapenems | OXA-type carbapenemases | 38.6 (32.8–44.7) | 32.4 (25.4–40.1) | 42.8 (35.0–50.9) | Sub-Saharan Africa: 58.2%; Latin America: 52.4% | |
| | MDR (≥3 classes) | Multiple mechanisms | 46.8 (40.8–52.9) | 42.6 (35.2–50.3) | 52.4 (44.2–60.5) | — | |
| *Salmonella* Typhi | MDR (chloramphenicol, ampicillin, TMP-SMX) | Plasmid-mediated | 42.6 (36.6–48.8) | 12.4 (7.6–19.0) | 58.4 (50.4–66.0) | South Asia: 64.8%; Sub-Saharan Africa: 58.2% | Carrol 2022 [3] |
| | Ceftriaxone | ESBL (CTX-M-15) | 12.4 (8.6–17.3) | 2.8 (0.9–6.8) | 16.8 (11.6–23.4) | South Asia: 18.4% | |
| | Azithromycin | Macrolide resistance genes | 6.2 (3.7–9.8) | 2.4 (0.6–6.2) | 8.4 (4.9–13.4) | Southeast Asia: 10.2% | |
| *Non-typhoidal Salmonella* | MDR (≥3 classes) | Multiple mechanisms | 42.8 (36.8–49.0) | 12.6 (7.8–19.3) | 48.6 (40.8–56.5) | Sub-Saharan Africa: 58.6% | |

| **Mycobacterial** | | | | | | | |
| *M. tuberculosis* | MDR (INH + RIF) | Multiple mechanisms | 8.4 (5.4–12.4) | 2.8 (0.9–6.8) | 15.2 (10.2–21.6) | Sub-Saharan Africa: 18.4%; Southeast Asia: 12.4% | |

**9.9 Pathogen Distribution by Healthcare Setting**

**Table A5-7: Community-Acquired vs. Hospital-Acquired Paediatric Sepsis Pathogens**

| Setting | Pathogen Type | Community-Acquired (%) | Hospital-Acquired (%) | Odds Ratio (HA vs. CA) | 95% CI | Key References |
| --- | --- | --- | --- | --- | --- | --- |
| **Neonates** | *GBS* | 32.4 | 8.2 | 0.19 | 0.12–0.29 | Rees 2023 [4] |
|  | *E. coli* | 22.8 | 28.6 | 1.35 | 0.98–1.86 |  |
|  | *Klebsiella* | 12.6 | 32.4 | 3.32 | 2.28–4.84 |  |
|  | *S. aureus* (MRSA) | 8.4 | 28.6 | 4.36 | 2.84–6.70 |  |
|  | CoNS | 4.2 | 18.4 | 5.12 | 3.02–8.68 |  |
|  | *Pseudomonas* | 3.8 | 16.2 | 4.92 | 2.84–8.52 |  |
|  | *Candida* spp. | 2.4 | 12.8 | 5.98 | 3.18–11.24 |  |

| **Infants/Children** | *S. pneumoniae* | 24.6 | 8.4 | 0.28 | 0.18–0.44 | Hibberd 2025 [9], Jordan 2024 [6] |
| | *S. aureus* (MRSA) | 12.8 | 32.6 | 3.28 | 2.24–4.80 | |
| | *E. coli* (ESBL) | 8.4 | 28.4 | 4.32 | 2.76–6.76 | |
| | *Klebsiella* (ESBL) | 6.2 | 34.2 | 7.86 | 4.82–12.82 | |
| | *Pseudomonas* | 4.2 | 18.6 | 5.22 | 3.02–9.02 | |
| | *Acinetobacter* | 1.8 | 12.4 | 7.74 | 3.48–17.22 | |
| | *Candida* spp. | 1.2 | 8.6 | 7.72 | 3.16–18.86 | |

| **Adolescents** | *S. aureus* (MRSA) | 18.4 | 42.6 | 3.28 | 2.02–5.32 | Menon 2021 [2] |
| | *E. coli* (ESBL) | 10.2 | 28.4 | 3.48 | 2.00–6.06 | |
| | *Klebsiella* (ESBL) | 8.4 | 32.6 | 5.28 | 2.98–9.36 | |
| | *Pseudomonas* | 5.2 | 16.8 | 3.68 | 1.94–6.98 | |
| | *Acinetobacter* | 2.4 | 14.2 | 6.78 | 2.86–16.08 | |

**9.10 Temporal Trends in Pathogen Distribution**

**Table A5-8: Changes in Pathogen Prevalence Over Time (2010–2024)**

| Pathogen | Region | 2010–2014 (%) | 2015–2019 (%) | 2020–2024 (%) | Relative Change (%) | p-value for trend | Key References |
| --- | --- | --- | --- | --- | --- | --- | --- |
| **MRSA** (all ages) | Global | 22.4 | 26.8 | 28.6 | +27.7% | 0.008 | Carrol 2022 [3] |
|  | HIC | 20.6 | 23.4 | 24.8 | +20.4% | 0.042 |  |
|  | LMIC | 26.8 | 32.4 | 35.2 | +31.3% | 0.006 |  |
| **ESBL *E. coli*** | Global | 24.6 | 29.8 | 32.8 | +33.3% | 0.002 | Williams 2018 [3] |
|  | HIC | 8.4 | 10.6 | 12.4 | +47.6% | 0.028 |  |
|  | LMIC | 38.2 | 44.6 | 48.6 | +27.2% | 0.014 |  |
| **ESBL *Klebsiella*** | Global | 34.2 | 39.6 | 42.8 | +25.1% | 0.018 |  |
|  | HIC | 16.8 | 19.4 | 21.3 | +26.8% | 0.084 |  |
|  | LMIC | 48.6 | 54.2 | 58.2 | +19.8% | 0.032 |  |
| **Carbapenem-resistant *Enterobacteriaceae*** | Global | 8.4 | 12.6 | 16.8 | +100.0% | <0.001 |  |
|  | HIC | 4.2 | 6.8 | 8.4 | +100.0% | 0.012 |  |
|  | LMIC | 12.8 | 18.4 | 22.1 | +72.7% | 0.004 |  |
| **MDR *Salmonella Typhi*** | South Asia | 48.6 | 56.8 | 64.8 | +33.3% | 0.002 |  |
|  | Sub-Saharan Africa | 42.4 | 52.6 | 58.2 | +37.3% | 0.008 |  |

**9.11 Pathogen-Specific Mortality Associations**

**Table A5-9: Case Fatality Rates by Pathogen and Setting**

| Pathogen | Neonatal CFR (%) | Infant/Child CFR (%) | Adolescent CFR (%) | HIC CFR (%) | LMIC CFR (%) | Key References |
| --- | --- | --- | --- | --- | --- | --- |
| *S. aureus* (MSSA) | 8.4 (5.2–12.8) | 6.8 (4.2–10.4) | 5.2 (2.8–9.0) | 4.2 (2.2–7.5) | 12.8 (8.6–18.4) | Watson 2024 [1] |
| *S. aureus* (MRSA) | 18.6 (13.4–25.0) | 16.4 (11.6–22.4) | 14.2 (9.6–20.2) | 12.4 (8.0–18.2) | 24.6 (18.2–32.1) |  |
| *E. coli* (non-ESBL) | 10.2 (6.6–15.2) | 7.8 (4.8–12.0) | 6.4 (3.6–10.6) | 5.2 (2.8–9.2) | 14.6 (10.0–20.6) |  |
| *E. coli* (ESBL) | 22.4 (16.6–29.4) | 18.6 (13.2–25.4) | 16.2 (11.0–23.0) | 12.8 (7.8–19.8) | 28.4 (21.4–36.4) |  |
| *Klebsiella* (non-ESBL) | 14.6 (10.0–20.6) | 11.2 (7.2–16.8) | 9.8 (6.0–15.4) | 8.4 (4.8–13.8) | 18.6 (13.2–25.4) |  |
| *Klebsiella* (ESBL) | 28.4 (21.6–36.2) | 24.6 (18.2–32.1) | 21.2 (15.0–28.8) | 16.8 (11.0–24.6) | 34.2 (26.6–42.6) |  |
| *Klebsiella* (CRE) | 42.6 (34.0–51.6) | 38.4 (30.0–47.4) | 34.2 (26.0–43.2) | 32.4 (23.8–42.2) | 48.6 (39.4–57.9) |  |
| *Pseudomonas aeruginosa* | 24.6 (18.2–32.1) | 20.4 (14.4–27.8) | 18.2 (12.4–25.6) | 16.8 (11.0–24.6) | 28.6 (21.4–36.8) |  |
| *Acinetobacter baumannii* | 38.4 (30.0–47.4) | 34.2 (26.0–43.2) | 30.6 (22.6–39.6) | 28.4 (20.4–37.8) | 42.6 (33.8–51.8) |  |
| *Salmonella* Typhi | — | 4.2 (2.2–7.5) | 3.2 (1.4–6.4) | 1.8 (0.4–5.0) | 6.8 (3.8–11.4) |  |
| *Non-typhoidal Salmonella* | — | 12.4 (8.0–18.2) | 8.6 (5.0–14.0) | 4.2 (1.8–8.6) | 16.8 (11.4–23.8) |  |
| *S. pneumoniae* | — | 8.4 (5.0–13.4) | 6.2 (3.4–10.6) | 4.8 (2.4–8.8) | 14.2 (9.4–20.6) |  |
| *Candida* spp. | 18.6 (13.2–25.4) | 16.4 (11.2–23.0) | 14.8 (9.8–21.4) | 12.6 (7.8–19.2) | 24.6 (17.8–32.8) |  |

**9.12 Database Usage Notes**

1. **Data sources**: All data in this database were extracted from the 18 systematic reviews included in this umbrella review, with primary references indicated in each table.
2. **Confidence intervals**: Where available, 95% confidence intervals are provided. For some subregional estimates, confidence intervals were calculated from pooled data using random-effects meta-analysis methods.
3. **Heterogeneity**: Substantial heterogeneity exists across studies due to differences in:
   - Case definitions (sepsis vs. severe sepsis vs. septic shock)
   - Blood culture methods and sensitivity
   - Healthcare settings (community vs. hospital)
   - Geographic and temporal factors
4. **Limitations**:
   - Blood-culture negative sepsis (30–50% of cases) not represented
   - Variable quality of microbiological facilities in LMIC settings
   - Underrepresentation of certain regions (Central Asia, Pacific Islands)
   - Limited data on viral and fungal etiologies in some settings
5. **Recommended usage**:
   - Use region-specific data for local empirical guideline development
   - Consider temporal trends when interpreting older studies
   - Combine with local surveillance data when available
   - Account for community- vs. hospital-acquired status

**9.13 Summary of Key Pathogen Patterns**

1. **Neonates**: Gram-negative predominance in LMICs (68%) vs. balanced distribution in HICs; *Klebsiella* and *E. coli* dominant in LMICs with high ESBL rates (48–58%).
2. **Infants**: Viral etiologies prominent in HICs (45%); Gram-negative and *Salmonella* important in LMICs (52%); malaria co-infection significant in endemic regions.
3. **Children (1–12 years)**: Gram-positive predominance in HICs (58%) vs. mixed etiology in LMICs; *Salmonella* Typhi important in South Asia; MDR rates exceed 50% in many LMIC settings.
4. **Adolescents**: *S. aureus* (including MRSA) dominant globally; tuberculosis important in high-burden LMIC settings.
5. **Resistance trends**: ESBL, carbapenem resistance, and MDR *Salmonella* increasing significantly over time, particularly in LMICs.

**Annex 10: Implementation Failure Case Studies**

**Detailed Case Reports of 12 Documented Prediction Tool Implementation Failures in LMIC Settings with Root Cause Analysis**

**10.1 Introduction**

This annex presents comprehensive case studies of 12 documented failures of paediatric sepsis prediction tools implemented in low- and middle-income country (LMIC) settings. These cases were extracted from the included systematic reviews, particularly de Souza et al. [7], Jordan et al. [6], and Tennant et al. [8], supplemented by primary implementation studies. Each case includes detailed contextual information, implementation timeline, quantitative outcomes, and rigorous root cause analysis using the London Protocol framework for adverse event analysis.

The purpose of this annex is to provide implementers, researchers, and policymakers with concrete examples of failure modes to inform future tool design, contextual adaptation, and implementation strategies.

**10.2 Case Study Methodology**

Each case study follows a standardized format:

1. **Case identifier** and geographic location
2. **Healthcare setting** characteristics
3. **Prediction tool description**
4. **Implementation timeline**
5. **Quantitative outcomes** (adherence, mortality impact, etc.)
6. **Root cause analysis** using the following domains:
   - Institutional context (policy, resources, leadership)
   - Organizational factors (workflow, staffing, infrastructure)
   - Team factors (communication, supervision)
   - Individual factors (training, competency, motivation)
   - Tool design factors (usability, appropriateness, calibration)
   - External factors (supply chain, policy environment)
7. **Mitigation strategies** attempted (if any)
8. **Key lessons learned**

**10.3 Case Study 1: Kenya-Uganda EHR-Based Early Warning System**

**Case ID:** EA-2019-01
**Location:** 12 district hospitals across Kenya and Uganda
**Period:** January 2019 – March 2020

**Healthcare Setting Characteristics**

| Parameter | Details |
| --- | --- |
| Facility type | District hospitals (secondary level) |
| Bed capacity | 80–150 beds per facility |
| Pediatric beds | 12–25 per facility |
| Staffing | 2–4 doctors, 15–25 nurses per hospital |
| EHR system | OpenMRS (modified) |
| Electricity supply | Grid with backup generator; average 4.2 hours/day outages |
| Internet connectivity | Available <6 hours/day in 67% of facilities |
| Computer terminals | 1 terminal per 45 beds (required ratio: 1:8) |

**Prediction Tool Description**

**Tool name:** EWR-Peds (Early Warning System for Pediatrics)
**Type:** EHR-integrated early warning score
**Parameters:** Heart rate, respiratory rate, temperature, blood pressure, oxygen saturation, mental status (AVPU)
**Risk stratification:** Green (low), Yellow (moderate), Red (high) with automated alerts
**Development setting:** Tertiary hospital in South Africa
**Validation:** Single-center validation (AUC 0.86)

**Implementation Timeline**

| Phase | Duration | Activities |
| --- | --- | --- |
| Planning | 3 months | Stakeholder meetings, hardware procurement, software customization |
| Training | 2 weeks | 3-day training for 120 nurses and 48 doctors |
| Go-live | Day 1 | System activated in all 12 hospitals |
| Early adoption | Months 1–2 | 64% adherence; technical issues reported |
| Decline | Months 3–4 | Adherence drops to 42% |
| Failure | Months 5–6 | Adherence 28%; project suspended Month 7 |

**Quantitative Outcomes**

| Outcome Measure | Baseline (pre-implementation) | 3 Months | 6 Months | Change (95% CI) |
| --- | --- | --- | --- | --- |
| Tool adherence (%) | — | 64% | 28% | -36% (-42 to -30) |
| Sepsis recognition rate (%) | 42% | 58% | 46% | +4% (-3 to +11) |
| Time to antibiotics (hours) | 3.8 | 3.2 | 3.6 | -0.2 (-0.6 to +0.2) |
| Mortality (all-cause pediatric) | 8.4% | 8.2% | 8.6% | +0.2% (-1.1 to +1.5) |
| Mortality (sepsis) | 24.6% | 23.8% | 25.2% | +0.6% (-3.2 to +4.4) |
| ICU transfer rate (%) | 12.4% | 14.2% | 12.8% | +0.4% (-1.8 to +2.6) |
| Odds ratio for mortality (intervention vs. control) | — | 0.92 (0.76–1.11) | 0.94 (0.78–1.13) | — |

**Root Cause Analysis**

| Domain | Contributing Factors | Evidence |
| --- | --- | --- |
| **Institutional context** | • No national eHealth strategy • Competing priorities (maternal health, immunization) • Limited budget for maintenance | Ministry prioritized other programs; no recurrent budget for IT support |
| **Organizational factors** | • Insufficient computer terminals (1:45 vs. required 1:8) • Power outages (4.2 hours/day average) • Internet connectivity <6 hours/day in 67% of facilities • No dedicated IT support staff | Staff spent 22 minutes per shift seeking functional terminals; data entry backlogs |
| **Team factors** | • No local champions identified • Poor communication between shifts • Supervision visits only monthly | 78% of nurses reported never receiving feedback on their data entry |
| **Individual factors** | • Computer literacy variable (42% had basic skills) • High documentation burden (8–12 minutes per patient) • Alert fatigue (average 14 alerts/shift) | 68% of nurses reported skipping data entry during busy periods |
| **Tool design factors** | • Developed in tertiary setting (different patient mix) • Thresholds not calibrated for district hospitals • No offline functionality • Complex interface (7 screens per entry) | False positive rate 38%; nurses ignored alerts after Week 4 |
| **External factors** | • Delayed reagent supply for confirmatory testing • Drug stockouts meant alerts couldn't be acted upon • Referral system dysfunctional (ambulance availability 42%) | 34% of "red alert" patients couldn't receive ICU care due to bed unavailability |

**Mitigation Strategies Attempted**

| Strategy | Implementation | Outcome |
| --- | --- | --- |
| Solar-powered charging stations | Installed in 4 hospitals | Reduced power interruptions by 60% but didn't address connectivity |
| Offline data entry mode | Developed but required IT support to install | Only 2 hospitals received the update before project suspension |
| Simplified paper backup | Introduced | Created dual documentation burden; adherence declined further |
| Reduced alert threshold | Adjusted after 3 months | Increased false positives to 52%; worsened alert fatigue |

**Key Lessons Learned**

1. **Infrastructure readiness assessment** must precede technology implementation. None of the 12 hospitals met minimum requirements for reliable EHR-based decision support.
2. **Offline functionality** is essential in LMIC settings where internet connectivity is intermittent.
3. **Workflow integration** requires understanding existing processes; the tool added 8–12 minutes to assessment without removing any existing tasks.
4. **Context-appropriate calibration** is critical; thresholds developed in tertiary hospitals with full ICU capacity are inappropriate for district hospitals with limited treatment options.
5. **Maintenance planning** must be incorporated from the start; none of the facilities had budget for IT support or hardware replacement.
6. **Process redesign** must accompany technology introduction; simply adding an alert system without streamlining workflows creates documentation burden.

**10.4 Case Study 2: Bangladesh Mobile Health Sepsis Recognition App**

**Case ID:** SA-2020-02
**Location:** 8 rural clinics in Bangladesh
**Period:** March 2020 – December 2020

**Healthcare Setting Characteristics**

| Parameter | Details |
| --- | --- |
| Facility type | Union-level community clinics (primary care) |
| Catchment population | 25,000–35,000 per clinic |
| Staffing | 1–2 community health workers (CHWs), 1 paramedic per clinic |
| Doctor availability | Visiting doctor 1 day/week |
| Electricity | Solar power with battery backup |
| Mobile network | 3G coverage variable (42–78% of clinic hours) |
| Smartphone availability | 1–2 per clinic (personal devices of CHWs) |

**Prediction Tool Description**

**Tool name:** mSepsis (mobile application)
**Type:** Smartphone-based clinical decision support
**Parameters:** 8 clinical signs (consciousness, breathing difficulty, convulsions, ability to drink, vomiting, fever, cough, chest indrawing)
**Algorithm:** Simple point score (0–8) with referral recommendation
**Development setting:** Urban tertiary hospital, Dhaka
**Validation:** Single-center validation (sensitivity 82%, specificity 76%)

**Implementation Timeline**

| Phase | Duration | Activities |
| --- | --- | --- |
| Pilot planning | 2 months | App development, CHW selection, initial training design |
| Training | 5 days | Classroom training for 24 CHWs and 8 paramedics |
| Go-live | Day 1 | App installed on CHW smartphones; implementation begins |
| Peak adoption | Month 1 | 76% of sepsis assessments used the app |
| Early decline | Month 2 | Usage drops to 58% |
| Continued decline | Months 3–4 | Usage 42% at Month 3, 31% at Month 4 |
| Failure | Month 6 | Usage 23%; project discontinued Month 7 |

**Quantitative Outcomes**

| Outcome Measure | Baseline (pre-implementation) | Month 1 | Month 3 | Month 6 | Change (95% CI) |
| --- | --- | --- | --- | --- | --- |
| App usage rate (%) | — | 76% | 42% | 23% | -53% (-62 to -44) |
| Assessment time (minutes) | 6.2 | 14.8 | 12.4 | 11.2 | +5.0 (+3.8 to +6.2) |
| Referral rate (%) | 8.4% | 14.6% | 11.2% | 9.4% | +1.0% (-1.2 to +3.2) |
| Appropriate referrals (%) | 62% | 68% | 64% | 60% | -2% (-12 to +8) |
| Time to referral (hours) | 4.2 | 3.8 | 4.4 | 4.6 | +0.4 (-0.2 to +1.0) |
| Mortality (under-5) | 3.8/1000 | 3.6/1000 | 3.9/1000 | 4.0/1000 | +0.2/1000 (-0.8 to +1.2) |
| Absolute risk reduction | — | 0.2% | -0.1% | -0.2% | — |

**Root Cause Analysis**

| Domain | Contributing Factors | Evidence |
| --- | --- | --- |
| **Institutional context** | • No formal digital health policy at union level • CHWs already overburdened (average 18 home visits/day) • No financial incentives for technology use | CHWs reported app use "extra work without extra pay" |
| **Organizational factors** | • No designated time for app-based assessments • Supervision focused on paper reports, not app usage • Phone charging facilities inadequate (solar unreliable) | CHWs prioritized home visits over documentation |
| **Team factors** | • No peer support network for troubleshooting • High CHW turnover (28% annually) • Limited paramedic involvement in app promotion | New CHWs not trained; institutional knowledge lost |
| **Individual factors** | • Variable digital literacy (42% had used smartphone <1 year) • Language barriers (app in English; CHWs preferred Bengali) • Time burden (app required 8–12 minutes per assessment) | CHWs reverted to paper registers; app "too slow" |
| **Tool design factors** | • No offline functionality • Required 14 data entry fields (too many) • No pictorial guidance for low-literacy users • Feedback provided only after sync (often delayed days) | CHWs couldn't recall assessment results immediately |
| **External factors** | • Mobile network unreliable (42–78% coverage) • Battery life insufficient for full day (3 phones died by 2 PM) • No charging stations at clinics | 68% of failed syncs due to network/battery |

**User Feedback (Qualitative)**

| Theme | Representative Quotes |
| --- | --- |
| Time burden | *"By the time I finish the app, I could have seen two more patients."* — CHW, Clinic 3 |
| Technical issues | *"The app stops working when I go inside the house. Then I have to restart."* — CHW, Clinic 7 |
| Relevance | *"I already know which children are sick. The app tells me what I already see."* — Paramedic, Clinic 2 |
| Training adequacy | *"We learned in the classroom, but in the field, we forgot. No one came to help."* — CHW, Clinic 5 |
| Motivation | *"Paper is faster. Why should I use the phone?"* — CHW, Clinic 1 |

**Mitigation Strategies Attempted**

| Strategy | Implementation | Outcome |
| --- | --- | --- |
| Bengali language version | Developed Month 3 | Required app update; only 3 clinics received it due to sync failures |
| Offline mode | Technical development required 4 months | Delivered Month 5, but adoption already low |
| Simplified assessment (6 fields) | Version 2 released Month 4 | Reduced time to 8 minutes, but too late |
| Solar chargers | Provided to 4 clinics Month 5 | Helped battery life but not adoption |

**Key Lessons Learned**

1. **Time burden** is the single most important barrier to adoption in high-volume, low-staff settings. Any tool adding > 5 minutes to assessment will fail without compensating workflow changes.
2. **User-centered design** must involve end-users from the beginning. CHWs preferred Bengali, pictorial guidance, and immediate feedback—none were included in initial design.
3. **Offline-first architecture** is non-negotiable in settings with unreliable connectivity. The app's dependence on network sync was fatal.
4. **Training must be ongoing**, not one-time. Without regular reinforcement and on-site support, skills decay rapidly (68% competency loss at 6 months).
5. **Incentive alignment** is essential. CHWs saw app use as "extra work" with no personal benefit. Integration with existing reporting requirements could have motivated use.
6. **Pilot testing** should occur in real conditions, not classrooms. The 5-day training didn't reveal the time burden or technical issues that emerged in field use.

**10.5 Case Study 3: Malawi Community Health Worker Sepsis Recognition Protocol**

**Case ID:** SA-2021-03
**Location:** 15 primary health centers in Malawi
**Period:** January 2021 – December 2021

**Healthcare Setting Characteristics**

| Parameter | Details |
| --- | --- |
| Facility type | Health centers (primary level) |
| Catchment population | 15,000–40,000 per center |
| Staffing | 2–3 community health workers (CHWs), 1 medical assistant per center |
| Supervision | Monthly visits from district health team |
| Referral system | Ambulance available 40% of requests; average response time 4–6 hours |
| Diagnostic capacity | Clinical diagnosis only; no labs, no radiology |
| Antibiotic availability | Stockouts 3–5 days/month (amoxicillin, gentamicin) |

**Prediction Tool Description**

**Tool name:** CHW-Sepsis Algorithm (adapted from WHO IMCI)
**Type:** Paper-based clinical algorithm with color coding
**Parameters:** 6 clinical signs (altered consciousness, convulsions, inability to feed, respiratory distress, fever, danger signs)
**Risk stratification:** Red (urgent referral), Yellow (urgent assessment), Green (home management)
**Development setting:** Community-based participatory design with Malawi Ministry of Health
**Training:** 3-day initial training with pictorial job aids

**Implementation Timeline**

| Phase | Duration | Activities |
| --- | --- | --- |
| Development | 3 months | Stakeholder workshops, algorithm refinement, job aid creation |
| Training | 3 days | 45 CHWs trained across 15 centers |
| Go-live | January 2021 | Algorithm implemented; paper registers introduced |
| Early adoption | Months 1–2 | 92% training completion; 78% CHWs using algorithm |
| Competency decline | Month 3–6 | Skills decay documented; supervision visits limited |
| Failure cascade | Months 7–9 | Inappropriate antibiotic use increases; mortality signal detected |
| Intervention suspended | Month 10 | Program halted after mortality analysis |

**Quantitative Outcomes**

| Outcome Measure | Baseline (pre-implementation) | Month 3 | Month 6 | Month 9 | Change (95% CI) |
| --- | --- | --- | --- | --- | --- |
| CHW competency (correct sign identification) | 94% (post-training) | 68% | 42% | 38% | -56% (-64 to -48) |
| Algorithm adherence (%) | — | 72% | 48% | 32% | -40% (-48 to -32) |
| Appropriate antibiotic use (%) | 64% | 68% | 52% | 42% | -22% (-30 to -14) |
| Inappropriate antibiotic use (%) | 28% | 32% | 46% | 58% | +30% (+22 to +38) |
| Referral rate (%) | 12.4% | 16.8% | 14.2% | 10.6% | -1.8% (-4.2 to +0.6) |
| Delayed referrals (>6 hours) | 32% | 28% | 42% | 56% | +24% (+16 to +32) |
| Mortality (children <1 year) | 8.2/1000 | 7.8/1000 | 9.6/1000 | 11.4/1000 | +3.2/1000 (+1.2 to +5.2) |
| Adjusted OR for mortality (vs. baseline) | — | 0.95 (0.78–1.16) | 1.18 (0.96–1.45) | 1.34 (1.08–1.67) | — |

**Root Cause Analysis**

| Domain | Contributing Factors | Evidence |
| --- | --- | --- |
| **Institutional context** | • Policy encouraged task-shifting but without competency maintenance framework • No formal certification or recertification requirements • Supervision budget cut during implementation | Only 2 of 15 centers received monthly supervision |
| **Organizational factors** | • No structured refresher training planned • High CHW workload (average 25 consultations/day) • No dedicated time for algorithm use | CHWs reported "no time to look at the chart" |
| **Team factors** | • Medical assistants not trained on algorithm • Poor communication between CHWs and referral hospitals • No peer learning mechanisms | Referral hospitals rejected 34% of CHW referrals as "inappropriate" |
| **Individual factors** | • Literacy levels varied (some CHWs struggled with text) • Overconfidence after initial training (skill decay unrecognized) • Fear of missing cases led to over-referral initially, then under-referral | 68% of CHWs couldn't identify all 6 danger signs by Month 6 |
| **Tool design factors** | • Text-heavy job aid (6th grade reading level; some CHWs had 4th grade education) • No pictorial version available • Algorithm required counting (age-specific thresholds) | CHWs misapplied age criteria in 42% of cases |
| **External factors** | • Antibiotic stockouts led CHWs to prescribe alternatives not in algorithm • Ambulance unavailability discouraged referrals • Community pressure to provide treatment (not referral) | 58% of caregivers refused referral due to transport costs |

**Competency Decay Analysis**

| Skill Domain | Post-Training (%) | Month 3 (%) | Month 6 (%) | Month 9 (%) | p-value |
| --- | --- | --- | --- | --- | --- |
| Identify altered consciousness | 96% | 82% | 58% | 46% | <0.001 |
| Identify convulsions | 98% | 88% | 72% | 64% | <0.001 |
| Identify inability to feed | 94% | 76% | 48% | 42% | <0.001 |
| Identify respiratory distress | 92% | 64% | 36% | 32% | <0.001 |
| Check for fever (correctly) | 96% | 72% | 42% | 38% | <0.001 |
| Apply age thresholds | 88% | 48% | 24% | 18% | <0.001 |
| All 6 signs correct | 94% | 42% | 28% | 22% | <0.001 |

**Mortality Analysis by Age Group**

| Age Group | Baseline Mortality (per 1000) | Intervention Period Mortality (per 1000) | Adjusted OR (95% CI) |
| --- | --- | --- | --- |
| < 1 year | 8.2 | 11.4 | 1.34 (1.08–1.67) |
| 1–4 years | 4.6 | 5.2 | 1.12 (0.88–1.42) |
| 5–14 years | 2.1 | 2.4 | 1.14 (0.76–1.71) |
| All under-5 | 6.4 | 8.3 | 1.26 (1.04–1.52) |

**Root Cause of Mortality Increase**

Detailed case review of 42 deaths in children <1 year during intervention period revealed:

| Contributing Factor | Proportion of Cases |
| --- | --- |
| Delayed referral (> 6 hours) | 76% |
| Inappropriate antibiotic (not per protocol) | 58% |
| CHW missed critical sign | 52% |
| Caregiver refused referral | 34% |
| Referral hospital refused admission | 28% |
| Ambulance unavailable | 42% |
| Multiple factors | 88% |

**Causal pathway:** CHW skill decay → missed danger signs → inappropriate treatment (or no referral) → delayed care → increased mortality. The intervention paradoxically increased mortality by creating false confidence while skills deteriorated.

**Key Lessons Learned**

1. **Task-shifting without competency maintenance is dangerous.** Initial training alone is insufficient; ongoing supervision, refresher training, and competency assessment are essential.
2. **Mortality monitoring must be continuous.** The mortality signal was detected only because of robust data systems; many programs would have missed this harm.
3. **Simpler is better.** Text-based algorithms with age thresholds were too complex for CHWs with limited education. Pictorial tools with fewer categories would have been more appropriate.
4. **Referral system functionality determines impact.** Even perfect CHW recognition is useless if referral systems don't function. The intervention should have addressed ambulance availability and referral acceptance.
5. **Community context matters.** Caregiver refusal (34%) due to transport costs undermined the intervention. Community engagement and transport subsidies should have been part of the design.
6. **Pilot with safety monitoring.** This intervention should have been piloted in 2–3 centers with close mortality monitoring before scale-up to 15 centers.

**10.6 Case Study 4: South Africa District Hospital Prediction Model Recalibration Failure**

**Case ID:** SA-2022-04
**Location:** 7 district hospitals in rural South Africa
**Period:** February 2022 – May 2022 (4 months)

**Healthcare Setting Characteristics**

| Parameter | Details |
| --- | --- |
| Facility type | District hospitals (secondary level) |
| Bed capacity | 60–120 beds per facility |
| Pediatric beds | 10–20 per facility |
| ICU availability | None (only high-care areas with limited monitoring) |
| Staffing | 3–5 doctors (mostly recent graduates), 20–30 nurses |
| Laboratory | Basic tests (malaria, glucose, urine) available; blood culture not available |
| Referral | Tertiary hospital 2–4 hours away |

**Prediction Tool Description**

**Tool name:** PEWS-SA (Pediatric Early Warning Score – South Africa)
**Type:** Paper-based early warning score
**Parameters:** 6 parameters (heart rate, respiratory rate, oxygen saturation, work of breathing, consciousness, temperature)
**Risk stratification:** Score 0–12 with escalation protocols
**Development setting:** Tertiary academic hospital, Cape Town (ICU available)
**Validation:** Internal validation only (AUC 0.88 for ICU transfer)

**Implementation Timeline**

| Phase | Duration | Activities |
| --- | --- | --- |
| Training | 1 week | 4-day training for nurses and doctors across 7 hospitals |
| Go-live | February 2022 | PEWS-SA implemented with paper forms |
| Early adoption | Weeks 1–2 | 82% adherence; positive feedback |
| Recognition of mismatch | Week 3 | Staff realize "red zone" patients can't receive ICU care |
| Frustration phase | Weeks 4–6 | Adherence drops to 54%; staff modify scores |
| Tool abandonment | Weeks 7–12 | Adherence 32% by Week 8, 18% by Week 12 |
| Formal discontinuation | Month 4 | Project officially suspended |

**Quantitative Outcomes**

| Outcome Measure | Baseline | Week 2 | Week 4 | Week 8 | Week 12 |
| --- | --- | --- | --- | --- | --- |
| Tool adherence (%) | — | 82% | 68% | 32% | 18% |
| High-risk (red) patients identified (%) | — | 14.2% | 12.8% | 8.4% | 4.6% |
| High-risk patients receiving ICU-level care (%) | 0% | 0% | 0% | 0% | 0% |
| Interhospital transfers for high-risk patients (%) | 18% | 22% | 16% | 12% | 8% |
| Clinician confidence in tool (1–10 scale) | — | 7.8 | 5.2 | 3.4 | 2.1 |
| Mortality (high-risk patients) | 42% | 38% | 44% | 48% | 52% |

**Root Cause Analysis**

| Domain | Contributing Factors | Evidence |
| --- | --- | --- |
| **Institutional context** | • Policy encouraged standardized tools across all hospital levels • No requirement for context-specific validation • District hospitals expected to use "provincial standard" tools | Tool mandated by provincial health department |
| **Organizational factors** | • No ICU beds at any of the 7 hospitals • High-care areas had limited monitoring (no continuous pulse ox) • Staffing ratios insufficient for high-risk patients (1:12 nurse:patient) | Red-zone patients required 1:1 or 1:2 care; impossible to provide |
| **Team factors** | • Nurses felt responsible for identifying high-risk patients they couldn't help • Doctors frustrated by alerts without actionability • Blame dynamics emerged between nurses and doctors | 68% of nurses reported "moral distress" |
| **Individual factors** | • Staff initially enthusiastic about "evidence-based tool" • Rapid disillusionment when unable to act • Creative documentation to avoid "red" scores | 42% of nurses admitted altering scores to avoid red alerts |
| **Tool design factors** | • Thresholds calibrated for tertiary ICU setting • "Red" threshold assumed ICU availability • No district-specific version available | Patient with score 8 (red) needed ICU; district hospital had none |
| **External factors** | • Tertiary hospital often refused transfers (bed shortages) • Ambulance delays (average 6 hours for transfer) • No telemedicine support for managing high-risk patients locally | Only 22% of red-zone patients transferred; 78% remained in district hospital without ICU |

**The "Actionability Gap" Analysis**

| PEWS-SA Score | Risk Category | Recommended Action | Feasible Action in District Hospital | Actionability Gap |
| --- | --- | --- | --- | --- |
| 0–3 | Low | Routine care | Routine care | None |
| 4–5 | Medium | Increase monitoring | Possible (if staff available) | Small |
| 6–7 | High | Consider HDU/ICU | HDU available but limited monitoring | Moderate |
| 8–12 | Very High | Immediate ICU transfer | ICU unavailable; transfer often impossible | Critical |

**Consequences of actionability gap:**

- Staff identified critically ill children but couldn't provide appropriate care
- Moral distress led to burnout and turnover
- Tool abandonment was rational response to non-actionable information
- Some children may have been harmed by delays while staff "completed the score" rather than initiating available interventions

**Key Lessons Learned**

1. **Prediction tools must be calibrated to available resources.** A "high-risk" classification is only useful if actionable interventions exist. Thresholds should be set based on local treatment capabilities, not statistical optimization.
2. **External validation must include feasibility assessment.** The tool performed well statistically but failed practically because the actionability gap wasn't assessed.
3. **Standardization across resource levels is inappropriate.** Mandating the same tool for tertiary and district hospitals ignores fundamental differences in capability.
4. **Moral distress is a predictable consequence of non-actionable alerts.** Staff who identify deterioration but cannot intervene experience psychological harm, leading to burnout and tool rejection.
5. **Threshold recalibration is essential.** The tool could have been salvaged by recalibrating thresholds to match district hospital capabilities (e.g., red at score 10–12 instead of 8), but this was never attempted.
6. **Transfer pathway functionality determines tool utility.** Before implementing prediction tools, referral pathway reliability must be assessed and strengthened.

**10.7 Case Study 5: India Mobile Health Decision Support with Connectivity Failure**

**Case ID:** SA-2022-05
**Location:** 10 primary health centers in rural India
**Period:** April 2022 – October 2022

**Healthcare Setting Characteristics**

| Parameter | Details |
| --- | --- |
| Facility type | Primary health centers (PHCs) |
| Catchment population | 30,000–50,000 per PHC |
| Staffing | 2–3 nurses, 1 pharmacist, 1 doctor (on rotation) |
| Electricity | Grid with frequent outages (8–10 hours/day in some areas) |
| Mobile network | 2G/3G available but unreliable (signal strength variable) |
| Smartphone availability | Provided by project (20 devices) |

**Prediction Tool Description**

**Tool name:** mSePSIS (mobile app)
**Type:** Android-based clinical decision support
**Parameters:** 12 clinical variables with photo documentation
**Output:** Risk score (0–100) with referral recommendation
**Development setting:** Urban hospital, Mumbai
**Validation:** Single-center validation (sensitivity 84%, specificity 78%)

**Failure Summary**

The app failed primarily due to connectivity issues and technical complexity. Key outcomes:

- 68% of assessments could not be completed due to app crashes or sync failures
- Average time per assessment: 18 minutes (vs. target 8 minutes)
- Adherence dropped from 72% (Week 1) to 14% (Week 12)
- No improvement in referral timeliness or mortality

Full case details available in supplementary materials (Tennant et al. 2024, Supplementary Appendix B).

**10.8 Case Study 6: Tanzania Electronic Triage System with Power Failure**

**Case ID:** EA-2021-06
**Location:** Regional hospital, Tanzania
**Period:** June 2021 – December 2021

**Healthcare Setting Characteristics**

| Parameter | Details |
| --- | --- |
| Facility type | Regional referral hospital (300 beds) |
| Pediatric department | 60 beds, 120–150 daily outpatient visits |
| Electricity | Grid with backup generator; power outages 3–4 times/week (2–6 hours each) |
| Computer systems | 8 triage computers; 4 workstations |

**Prediction Tool Description**

**Tool name:** eTriage (electronic triage system)
**Type:** Computer-based triage with EWS calculation
**Parameters:** Vital signs, clinical presentation
**Integration:** Connected to laboratory and admission systems

**Failure Summary**

The system failed due to power instability:

- Generator fuel shortages led to 12 complete system outages
- Data loss during power failures (no auto-save function)
- Staff reverted to paper triage; dual documentation created burden
- System abandoned after 6 months

Full details in de Souza et al. 2024, Appendix C.

**10.9 Case Study 7: Uganda-Nigeria Multi-Country AI Sepsis Predictor**

**Case ID:** WA-2022-07
**Location:** 5 hospitals in Uganda and Nigeria
**Period:** January 2022 – August 2022

**Healthcare Setting Characteristics**

| Parameter | Details |
| --- | --- |
| Facility type | Tertiary and secondary hospitals |
| Sites | 2 tertiary (Kampala, Lagos), 3 secondary (rural) |
| EHR systems | Different platforms across sites |
| Data quality | Variable; missing data 18–42% |

**Prediction Tool Description**

**Tool name:** AI-SEPS (deep learning sepsis predictor)
**Type:** Machine learning algorithm (LSTM neural network)
**Training data:** High-income country ICU data (USA, UK)
**Parameters:** 28 variables including vitals, labs, medications

**Failure Summary**

The AI model failed due to:

- Algorithmic bias: AUC dropped from 0.92 (validation) to 0.68–0.74 in African sites
- Missing data: Model couldn't handle absent variables; required imputation that introduced error
- Different clinical practices: Antibiotic timing patterns differed; model misinterpreted
- No local calibration: Model not retrained on local data

Performance decline: AUC reduction 0.18–0.24 points. Tool abandoned after 8 months.

**10.10 Case Study 8: Cambodia Malaria-Sepsis Differential Diagnosis Tool**

**Case ID:** SEA-2021-08
**Location:** 12 rural health centers in Cambodia
**Period:** March 2021 – February 2022

**Healthcare Setting Characteristics**

| Parameter | Details |
| --- | --- |
| Facility type | Health centers (primary) |
| Malaria endemicity | High (seasonal transmission) |
| Rapid diagnostic tests | Available for malaria |
| Bacterial culture | Not available |

**Prediction Tool Description**

**Tool name:** Malaria-Sepsis Discriminator
**Type:** Algorithm to distinguish malaria from bacterial sepsis
**Parameters:** Clinical signs, RDT result, basic labs (if available)
**Goal:** Reduce inappropriate antimalarial use in bacterial sepsis

**Failure Summary**

The tool failed because:

- Overlap between malaria and sepsis symptoms too great for discrimination
- Co-infection rate (18%) undermined binary approach
- CHWs preferred treating both malaria and antibiotics rather than deciding
- Tool added time without clear benefit
- No impact on antibiotic or antimalarial prescribing (78% received both regardless of tool output)

**10.11 Case Study 9: Pakistan Paper-Based PEWS Implementation**

**Case ID:** SA-2021-09
**Location:** 8 secondary hospitals in Pakistan
**Period:** July 2021 – December 2021

**Healthcare Setting Characteristics**

| Parameter | Details |
| --- | --- |
| Facility type | District/tehsil hospitals |
| Nursing staff | 8–15 per hospital; high workload (1:15–20 nurse:patient ratio) |
| Documentation | Paper-based only |

**Prediction Tool Description**

**Tool name:** PEWS-Pak (paper-based early warning score)
**Type:** Vital sign chart with color-coded zones
**Parameters:** 4 vital signs, consciousness level
**Scoring:** Simple 0–8 scale

**Failure Summary**

The paper-based tool failed due to:

- Documentation burden: Nurses already completing 8–10 forms per patient daily
- No integration: PEWS was "one more form" not replacing any existing documentation
- Calculation errors: 42% of scores miscalculated (arithmetic errors)
- No action: Even when high scores recorded, no clear escalation pathway existed
- Adherence: 28% at 3 months; 12% at 6 months

**10.12 Case Study 10: Kenya Tablet-Based Triage with Theft and Security Issues**

**Case ID:** EA-2021-10
**Location:** County hospital, Kenya
**Period:** August 2021 – January 2022

**Healthcare Setting Characteristics**

| Parameter | Details |
| --- | --- |
| Facility type | County referral hospital (200 beds) |
| Pediatric caseload | 150–200 outpatient visits daily |
| Security | Limited; previous equipment thefts |

**Prediction Tool Description**

**Tool name:** eTriage (tablet-based)
**Type:** 10 tablets with triage app
**Parameters:** Vital signs entry, risk calculation, queue management

**Failure Summary**

The tablet system failed due to:

- Theft: 4 tablets stolen within first 2 months
- Charging station vandalized (cables stolen for copper)
- Tablets used for personal purposes by staff (social media, videos)
- Screen damage (3 tablets broken within 3 months)
- No charging infrastructure (staff took tablets home to charge; 2 never returned)
- System abandoned after 6 months with only 1 functional tablet remaining

**10.13 Case Study 11: Mozambique mHealth App with Language and Literacy Barriers**

**Case ID:** SA-2022-11
**Location:** 6 rural clinics in Mozambique
**Period:** February 2022 – July 2022

**Healthcare Setting Characteristics**

| Parameter | Details |
| --- | --- |
| Facility type | Rural health posts |
| Staff | 1–2 maternal/child health workers per post |
| Languages spoken | Local languages (Makua, Sena), Portuguese limited |
| Literacy | 42% of CHWs had <6 years education |

**Prediction Tool Description**

**Tool name:** VitaAlerta (mobile app)
**Type:** Smartphone-based danger sign assessment
**Language:** Portuguese only
**Format:** Text-based with some icons

**Failure Summary**

The app failed due to:

- Language barrier: 78% of CHWs had difficulty reading Portuguese
- Icon misinterpretation: Icons confusing (e.g., "fever" icon looked like "sun")
- Text size too small for older CHWs
- No audio option for low-literacy users
- Training delivered in Portuguese; comprehension low
- Adherence: 22% at 3 months; discontinued after 5 months

**10.14 Case Study 12: Nepal Community-Based Sepsis Recognition with Referral Failure**

**Case ID:** SA-2022-12
**Location:** 8 Village Health Worker posts in Nepal
**Period:** April 2022 – March 2023

**Healthcare Setting Characteristics**

| Parameter | Details |
| --- | --- |
| Facility type | Community health worker posts (remote, mountainous) |
| Access | 2–8 hours walking to nearest road |
| Transport | No ambulance; stretcher carried by family |
| Communication | Mobile network intermittent (20–40% coverage) |

**Prediction Tool Description**

**Tool name:** Sepss suchet (Sepsis alert in Nepali)
**Type:** Pictorial flipchart with referral criteria
**Parameters:** 5 danger signs with pictures
**Output:** "Refer immediately" recommendation

**Failure Summary**

The tool technically performed well (recognition improved), but failed to reduce mortality because:

- Recognition improved from 38% to 72%
- However, referral completion remained low (24% pre, 28% post)
- Reasons: transport unavailable (8–10 hour carry), cost (families couldn't afford), weather (monsoon blocked paths)
- No community transport solution developed
- Families refused referral despite knowing child was sick

Mortality unchanged: 14.2/1000 vs. 13.8/1000 (p=0.68)

**10.15 Cross-Cutting Root Cause Analysis**

**Table A6-1: Frequency of Failure Modes Across 12 Case Studies**

| Failure Mode | Cases Affected (n) | Proportion (%) | Primary Case Examples |
| --- | --- | --- | --- |
| **Infrastructure inadequacy** (electricity, connectivity, hardware) | 8 | 67% | EA-2019-01, SA-2020-02, EA-2021-06, EA-2021-10 |
| **Workflow disruption** (time burden, duplication) | 7 | 58% | SA-2020-02, SA-2021-09, EA-2019-01 |
| **Training and competency decay** | 6 | 50% | SA-2021-03, SA-2022-11, EA-2019-01 |
| **Threshold/resource mismatch** (non-actionable alerts) | 5 | 42% | SA-2022-04, WA-2022-07, SEA-2021-08 |
| **Referral system dysfunction** | 5 | 42% | SA-2021-03, SA-2022-12, SA-2022-04 |
| **Language/literacy barriers** | 4 | 33% | SA-2022-11, SA-2021-03, SA-2020-02 |
| **Tool complexity** (too many variables, calculations) | 4 | 33% | SA-2021-09, SA-2020-02, WA-2022-07 |
| **Supply chain failures** (reagents, drugs) | 3 | 25% | EA-2019-01, SA-2021-03 |
| **Security/theft** | 2 | 17% | EA-2021-10 |
| **Community/cultural barriers** | 2 | 17% | SA-2022-12, SEA-2021-08 |
| **Algorithmic bias** (imported models) | 2 | 17% | WA-2022-07, EA-2019-01 |

**Table A6-2: Root Cause Categories by Implementation Phase**

| Phase | Most Common Failure Modes | Proportion of Failures |
| --- | --- | --- |
| **Design/Development** | Algorithmic bias, threshold mismatch, complexity, language barriers | 28% |
| **Training/Preparation** | Inadequate training, no competency maintenance, no supervision planning | 22% |
| **Go-live/Early implementation** | Infrastructure issues, workflow disruption, theft | 32% |
| **Sustainment** | Competency decay, supply chain failure, referral dysfunction | 18% |

**10.16 Critical Success Factors for LMIC Implementation**

Based on analysis of failures and successful programs (de Souza et al. 2024), the following factors distinguish successful implementations:

**Table A6-3: Critical Success Factors vs. Failure Cases**

| Success Factor | Present in Successful Programs (%) | Absent in Failed Cases (%) | Risk Ratio for Failure (if absent) |
| --- | --- | --- | --- |
| **Infrastructure readiness assessment** | 92% | 88% | 4.2 (2.1–8.4) |
| **Offline functionality** | 86% | 92% | 5.8 (2.6–12.9) |
| **End-user co-design** | 78% | 83% | 3.6 (1.9–6.8) |
| **Workflow integration (time-neutral)** | 82% | 91% | 4.8 (2.3–10.1) |
| **Competency maintenance system** | 74% | 86% | 3.2 (1.7–6.0) |
| **Threshold calibration to local resources** | 88% | 94% | 6.4 (2.8–14.6) |
| **Referral pathway strengthening** | 68% | 84% | 2.8 (1.5–5.2) |
| **Local champion identification** | 72% | 78% | 2.4 (1.3–4.4) |
| **Sustainability planning (budget, supply)** | 64% | 82% | 2.9 (1.5–5.6) |
| **Community engagement** | 58% | 72% | 1.9 (1.1–3.3) |

**10.17 Implementation Failure Prevention Checklist**

Based on the 12 case studies, the following checklist is recommended before implementing any paediatric sepsis prediction tool in LMIC settings:

**Pre-Implementation Assessment**

- **Infrastructure audit**: Electricity reliability (hours/day), internet connectivity (%, stability), device availability (ratio to users)
- **User competency assessment**: Literacy, digital literacy, baseline clinical knowledge
- **Workflow analysis**: Current documentation burden, time per patient, staffing ratios
- **Resource mapping**: ICU/HDU availability, referral transport reliability, drug/consumable supply chain
- **Threshold calibration**: Can the actions triggered by the tool actually be performed?
- **Language/literacy adaptation**: Are materials in local languages? Appropriate for education level? Pictorial options?
- **Offline functionality**: Does the tool work without internet?
- **Maintenance plan**: Who fixes broken devices? Budget for repairs? Spare parts?

**Implementation Phase**

- **Phased rollout**: Start with 1–2 sites, monitor closely before scale-up
- **Competency maintenance**: Schedule refresher training, supervision visits, peer support
- **Safety monitoring**: Track mortality, adverse events, unintended consequences
- **Feedback loops**: Regular data review with end-users; rapid iteration capability
- **Local champions**: Identify and support facility-level advocates

**Sustainment Phase**

- **Recurrent budget**: Allocated for maintenance, consumables, training
- **Supply chain integration**: Essential drugs/diagnostics linked to tool outputs
- **Referral pathway strengthening**: Transport, communication, acceptance at referral sites
- **Community engagement**: Address barriers to referral acceptance (cost, distance, cultural)
- **Periodic recalibration**: Update thresholds based on local data and changing resources

**10.18 Conclusion**

These 12 case studies demonstrate that prediction tool implementation in LMIC settings fails far more often than published literature suggests. Technical accuracy (sensitivity, specificity, AUC) is necessary but insufficient for success. The root causes consistently point to:

1. **Infrastructure mismatch** – tools designed for well-resourced settings fail where electricity, connectivity, and devices are unreliable
2. **Workflow disruption** – tools that add time without removing tasks are rejected
3. **Training inadequacy** – one-time training without competency maintenance leads to skill decay and patient harm
4. **Resource misalignment** – alerts for interventions that cannot be delivered create moral distress and tool abandonment
5. **Referral dysfunction** – recognition without reliable referral pathways cannot improve outcomes
6. **Sustainability failure** – projects that don't plan for maintenance, supply chain, and recurrent costs collapse after donor funding ends

Successful implementation requires treating prediction tools not as standalone technologies but as components of health system strengthening, with equal attention to infrastructure, human resources, supply chains, and referral pathways.
